# One-Step Hydrothermal Synthesis of Sn-Doped Sb_2_Se_3_ for Solar Hydrogen Production

**DOI:** 10.1021/acscatal.4c01762

**Published:** 2024-06-18

**Authors:** Zhenbin Wang, Sanghyun Bae, Miloš Baljozović, Pardis Adams, David Yong, Erin Service, Thomas Moehl, Wenzhe Niu, S. David Tilley

**Affiliations:** † Department of Chemistry, University of Zurich, Winterthurerstrasse 190, Zurich 8057, Switzerland; ‡ Molecular Surface Science Group, Empa, Swiss Federal Laboratories for Materials Science and Technology, Dübendorf 8600, Switzerland; § Laboratory of Photonics and Interfaces, Institute of Chemical Sciences and Engineering, École Polytechnique Fédérale de Lausanne, Lausanne 1015, Switzerland

**Keywords:** hydrothermal synthesis, Sb_2_Se_3_ photocathode, tin doping, surface passivation, carrier density

## Abstract

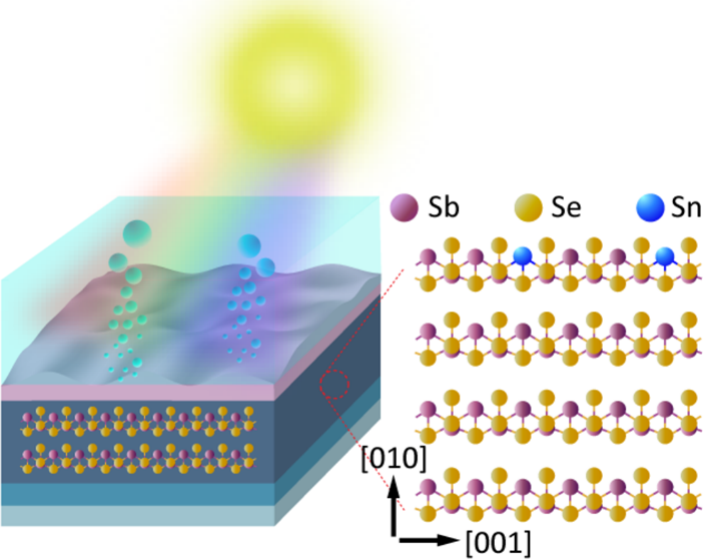

Antimony selenide (Sb_2_Se_3_) has recently been intensively investigated and has achieved significant advancement in photoelectrochemical (PEC) water splitting. In this work, a facile one-step hydrothermal method for the preparation of Sn-doped Sb_2_Se_3_ photocathodes with improved PEC performance was investigated. We present an in-depth study of the performance enhancement in Sn-doped Sb_2_Se_3_ photocathodes using capacitance–voltage (CV), drive-level capacitance profiling (DLCP), and electrochemical impedance spectroscopy (EIS) techniques. The incorporation of Sn^2+^ into the Sb_2_Se_3_ results in increased carrier density, reduced surface defects, and improved charge separation, thereby leading to improved PEC performance. With a thin Sb_2_Se_3_ absorber layer (270 nm thickness), the Sn-doped Sb_2_Se_3_ photocathode exhibits an improved photocurrent density of 17.1 mA cm^–2^ at 0 V versus RHE (*V*_RHE_) compared to that of the undoped Sb_2_Se_3_ photocathode (14.4 mA cm^–2^). This work not only highlights the positive influence of Sn doping on Sb_2_Se_3_ photocathodes but also showcases a one-step method to synthesize doped Sb_2_Se_3_ with improved optoelectronic properties.

## 1 Introduction

Photoelectrochemical (PEC) water splitting has been considered as a promising approach for producing renewable hydrogen from water and sunlight.^1^ To realize scalable hydrogen production with this approach, it is crucial to develop simple methods for the preparation of cost-effective semiconducting materials. Sb_2_Se_3_ has recently gained significant attention as a candidate for PEC water splitting and solar cells, benefiting from its excellent optoelectronic and electro(chemical) properties, such as high absorption coefficient (α > 10^5^ cm^–1^ in the near-infrared and visible region),^2^ suitable band gap (1.1–1.3 eV),^3^ good carrier mobility (∼10 cm^2^ V^–1^ s^–1^),^4^ low cost, and photocorrosion resistance.^5^ For PEC water splitting, the photocurrent density (*J*_ph_) of Sb_2_Se_3_ photocathodes has been extensively improved from 2 to 35.7 mA cm^–2^ at 0 V versus a reversible hydrogen electrode (*V*_RHE_), nearly approaching the maximum theoretical value of 40.9 mA cm^–2^.^6^ However, the photovoltage of Sb_2_Se_3_ photocathodes is still far behind the theoretical maximum value, attributed to a rich defect chemistry stemming from the two nonequivalent Sb and three nonequivalent Se atomic sites. The free carrier density of undoped Sb_2_Se_3_ determined from experimental data is only 10^13^ cm^–3^, considerably below the optimal doping density (10^16^ cm^–3^).^7,8^ These inherent properties pose a substantial challenge toward further improvement of the photovoltage.

Doping is an effective strategy for modifying physical and electronic properties by generating benign defects, improving conductivity, regulating crystallization, and suppressing surface or grain boundary defects. Various types of dopants including Na,^9^ Fe,^10^ K,^11^ Te,^12^ S,^13^ Bi,^14^ and Cu,^15,16^ have been extensively explored to improve the solar cell and PEC performance of Sb_2_Se_3_ photocathodes. For example, Te doping in Sb_2_Se_3_ solar cells, prepared through spin coating, has been shown to effectively suppress deep-level defects by regulating the atomic ratio of Se/Sb.^17^ The Ding group successfully mitigated surface defects and enhanced conductivity in Sb_2_Se_3_ through KOH etching treatment, resulting in a notable increase in the power conversion efficiency (PCE) of Sb_2_Se_3_ solar cells from 4.8 to 7.16%.^11^ Theoretical modeling has suggested that Sn, acting as an extrinsic substitution (Sn_Sb_), could improve the p-type conductivity of Sb_2_Se_3_.^18^ Recently, Liang et al. prepared Sn-doped Sb_2_Se_3_ solar cells by sputtering a Sn-doped Sb_2_Se_3_ target, suggesting enhanced p-type conductivity.^19^ In addition, Hobson et al. confirmed that Sn as a dopant enhances the p-type conductivity of Sb_2_Se_3_ using the Hall effect and hot-probe measurements.^20^

Various methods have been proposed for the fabrication of Sb_2_Se_3_, such as close-spaced sublimation,^3,21^ thermal evaporation,^22^ spin coating,^23^ electrodeposition,^24^ magnetron sputtering,^25^ and chemical bath deposition.^26^ In particular, the hydrothermal method offers precise control over nucleation and growth of inorganic thin films, which is regarded as a convenient and cost-effective strategy for fabricating thin films with low energy consumption. Currently, the most efficient Sb_2_Se_3_ solar cells have been achieved in superstrate configuration using a chemical bath deposition approach. With selenourea as an additive, the Sb_2_Se_3_ planar solar cells delivered a benchmark PCE of 10.57%.^26^ In 2021, Chen et al. developed a hydrothermal approach for superstrate Sb_2_Se_3_ solar cells, reaching a PCE of 7.9%.^27^ However, there has been relatively little attention on the hydrothermal synthesis of substrate-based Sb_2_Se_3_ solar cells and Sb_2_Se_3_ photocathodes for PEC water splitting, possibly because of challenges in nucleation on the substrate. Given the benchmark achievement in Sb_2_Se_3_ solar cells using a solution-processed strategy, developing a hydrothermal method for synthesizing substrate-based Sb_2_Se_3_ photocathodes becomes imperative.

Here, we propose a novel hydrothermal method for the synthesis of high-quality, compact, and phase-pure Sb_2_Se_3_ films in substrate configuration. By introducing Sn^2+^ cations into the precursor solution, surface defects of Sb_2_Se_3_ films are significantly suppressed, reducing from 2.67 × 10^12^ cm^–2^ (undoped Sb_2_Se_3_) to 8.64 × 10^11^ cm^–2^ (Sn-doped Sb_2_Se_3_). Meanwhile, the carrier density is increased in the Sn-doped Sb_2_Se_3_ film. These advantages of Sn doping result in greatly enhanced PEC performance with a notable improvement in the fill factor (FF). Importantly, the reproducibility of Sb_2_Se_3_ photocathodes is also slightly enhanced upon adding Sn^2+^ as a dopant.

## 2 Results and Discussion

### 2.1 Preparation of Sb_2_Se_3_ Films

The undoped and Sn-doped Sb_2_Se_3_ films supported on Mo-coated glass substrates were prepared using a simple hydrothermal method. This process involved fine-tuning precursor ratios between tin sulfate (SnSO_4_) and potassium antimony tartrate trihydrate (K_2_Sb_2_(C_4_H_2_O_6_)_2_·3H_2_O). The solution preparation process used for the hydrothermal synthesis of Sb_2_Se_3_ films is depicted in Figure S1 (Supporting Information) and described in the Experimental Section. The potassium antimony tartrate trihydrate and selenourea precursors were dissolved in Milli-Q water (18.2 MΩ·cm) serving as Sb and Se sources, respectively. To prevent the formation of elemental selenium (Se^0^), sodium sulfite (Na_2_SO_3_) was added into the aqueous solution to scavenge traces of oxygen.^28^ After the addition of sodium sulfite, a white precipitate was formed, primarily attributed to the increase in the solution’s pH from 4.13 to 6.89. To improve reproducibility and achieve high-quality Sb_2_Se_3_ films, the white precipitation was removed from the solution by filtering. Powder X-ray diffraction (PXRD) was used to characterize the crystallographic identity of the white powders. The observed diffraction peaks were well indexed to Sb_2_O_3_ (Figure S2). The Sb_2_Se_3_ films were obtained by placing Mo-coated glass substrates in the solution, followed by holding the reaction at 165 °C for different durations, as illustrated in Figure S3. The resulting film thicknesses for reaction times of 3, 4, and 5 h are shown in Figure S4 and are denoted as Sb_2_Se_3_-3h, Sb_2_Se_3_–Control, and Sb_2_Se_3_-5h, respectively. By adjusting the reaction duration, the thickness of the Sb_2_Se_3_ films was controlled, ranging from 200 to 400 nm. In this study, the optimal condition of 165 °C for 4 h was selected for further analysis (see details below). To further elucidate the impact of sodium sulfite, a Sb_2_Se_3_ film was prepared without its addition. Figure S5 exhibits the scanning electron microscopy (SEM) and X-ray diffraction (XRD) of this film. This film exhibits a nanowire structure that is unable to fully cover the Mo substrate, leading to a dark current during measurements. Furthermore, the XRD pattern indicates the formation of a secondary phase, Se^0^.

### 2.2 PEC Performance of Sb_2_Se_3_ Photocathodes

**Figure 1:**
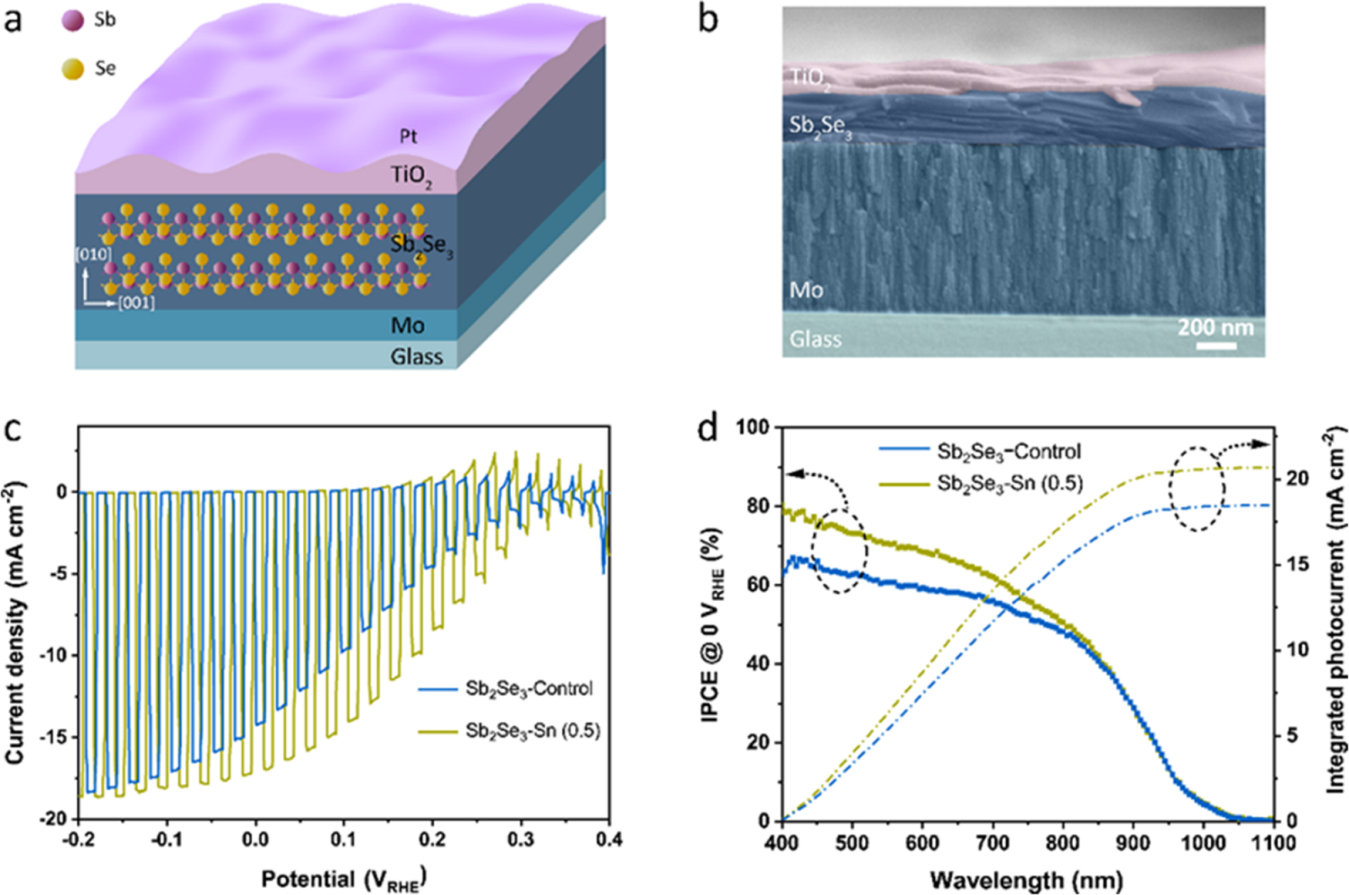
(a) Schematic illustration of the device configuration (Glass/Mo/Sb_2_Se_3_/TiO_2_/Pt). (b) The corresponding cross-sectional SEM image of the Sb_2_Se_3_ photocathode. (c) LSV plots of Sb_2_Se_3_–Control and Sb_2_Se_3_–Sn (0.5) photocathodes under intermittent simulated light illumination (AM 1.5 G, 100 mW cm^–2^), recorded with a scan rate of 10 mV s^–1^ in 1 M H_2_SO_4_ solution (pH = 0). (d) Incident photon-to-current conversion efficiency (IPCE) and integrated photocurrent density of both photocathodes at 0 *V*_RHE_ under 10% white light illumination.

A 30 nm TiO_2_ layer was deposited on Sb_2_Se_3_ films using atomic layer deposition (ALD). Although a TiO_2_ overlayer is often used for protecting unstable light absorber materials in PEC water splitting, the Sb_2_Se_3_ is resistant to photocorrosion^5^ and we use it here to construct a p–n junction, which promotes photogenerated electron–hole pair separation. Subsequently, 2 nm Pt, acting as a hydrogen evolution catalyst, was deposited by sputtering. The device configuration of the Sb_2_Se_3_ photocathode is shown in Figure 1a,b. The presence of TiO_2_ and Pt was confirmed by EDS measurement, as seen in Figure S6. The PEC performance of Sb_2_Se_3_ photocathodes prepared at different reaction durations (3–5 h) was compared (Figures 1c and S7). The Sb_2_Se_3_–Control photocathode exhibits a *J*_ph_ of 14.4 mA cm^–2^ at 0 *V*_RHE_, which is higher than that of the Sb_2_Se_3_-3h and Sb_2_Se_3_-5h photocathodes. For Sn-doped Sb_2_Se_3_ films, the molar ratios of Sn^2+^/Sb^3+^ prepared in the precursor solution were 0.5, 1, and 1.5%, respectively. Hereinafter, we refer to these films as Sb_2_Se_3_–Sn (0.5), Sb_2_Se_3_–Sn (1.0), and Sb_2_Se_3_–Sn (1.5). Figure 1c displays the PEC performance of undoped and Sn-doped Sb_2_Se_3_ photocathodes evaluated in a 1 M H_2_SO_4_ (pH = 0) electrolyte by the linear sweep voltammetry (LSV) method under intermittent light illumination (AM 1.5 G, 100 mW cm^–2^). An improvement in PEC performance in the Sb_2_Se_3_–Sn (0.5) photocathode, compared to the undoped Sb_2_Se_3_ photocathode, was observed. The champion device of the Sb_2_Se_3_–Sn (0.5) photocathode exhibits a *J*_ph_ of 17.1 mA cm^–2^ at 0 *V*_RHE_, which is higher than that of the Sb_2_Se_3_–Control photocathode. Furthermore, the Sb_2_Se_3_–Sn (0.5) photocathode also achieved a superior fill factor (FF) of 28.7%, in contrast to the 21.5% FF obtained by the Sb_2_Se_3_–Control. However, further increasing Sn doping concentrations, *J*_ph_ gradually decreased to 13.5 and 12 mA cm^–2^ at 0 *V*_RHE_ for the Sb_2_Se_3_–Sn (1.0) and Sb_2_Se_3_–Sn (1.5) photocathodes, respectively (Figure S8).

To gain insight into the PEC performance enhancement in the Sb_2_Se_3_–Sn (0.5) photocathode, incident photon-to-current conversion efficiency (IPCE) measurements were performed at 0 *V*_RHE_ in 1 M H_2_SO_4_ electrolyte under 10% white light illumination, as seen in Figure 1d. Both photocathodes show the same photoresponse onset (1050 nm), indicating the unchanged band gap with the Sn^2+^ doping treatment. The Sb_2_Se_3_–Sn (0.5) photocathode exhibits improved photoconversion efficiency across abroad wavelength range from 400 to 800 nm and reaches 80% of IPCE at 400 nm. In contrast, the IPCE value of the Sb_2_Se_3_–Control photocathode is below 67% in the whole wavelength range. The IPCE values of both Sb_2_Se_3_ photocathodes gradually decrease as the wavelength increases. Moreover, the IPCE values of both Sb_2_Se_3_ photocathodes at longer wavelengths (over 850 nm) are identical. This finding potentially implies reduced recombination near the Sb_2_Se_3_/TiO_2_ junction of the Sn-doped Sb_2_Se_3_ photocathode, as indicated by the higher IPCE in the blue region. Furthermore, the consistent IPCE in the longer-wavelength region is likely attributed to the thin Sb_2_Se_3_ films, which are unable to fully absorb all incident radiation (around 270 nm thickness, Figure S4). To fully absorb the photons at higher wavelengths, λ > 800 nm, an 800 nm thick Sb_2_Se_3_ thin film is required.^29^ Integrating the IPCE values with the AM 1.5 G spectrum yields a current density of 18.4 and 20.6 mA cm^–2^ for the Sb_2_Se_3_–Control and Sb_2_Se_3_–Sn (0.5) photocathodes, respectively. Both values are slightly higher than the *J*_ph_ obtained from the LSV measurements (Figure 1c) tested under 100% illumination of simulated solar light. The slight discrepancy in the integrated current is due to the light intensity dependence of the photocurrent. The *J*_ph_ of the Sb_2_Se_3_–Control and Sb_2_Se_3_–Sn (0.5) photocathodes at 0 *V*_RHE_ under different light intensities are depicted in Figure S9. Both Sb_2_Se_3_ photocathodes exhibit increased recombination and therefore more losses at higher light intensity, which explains the higher integrated photocurrent obtained from IPCE measurement under 10% white light illumination. IPCE measurement was also carried out at 0.15 *V*_RHE_. A considerable enhancement of the IPCE values in the wavelength range of 400–1050 nm was observed in the Sb_2_Se_3_–Sn (0.5) photocathode (Figure S10). The IPCE value for the Sb_2_Se_3_–Sn (0.5) photocathode reaches 65% at the 400 nm excitation wavelength, which is twice as high as that of the Sb_2_Se_3_–Control photocathode. This improvement indicates the increased light harvesting efficiency near the front surface of the Sb_2_Se_3_ absorber.

**Figure 2:**
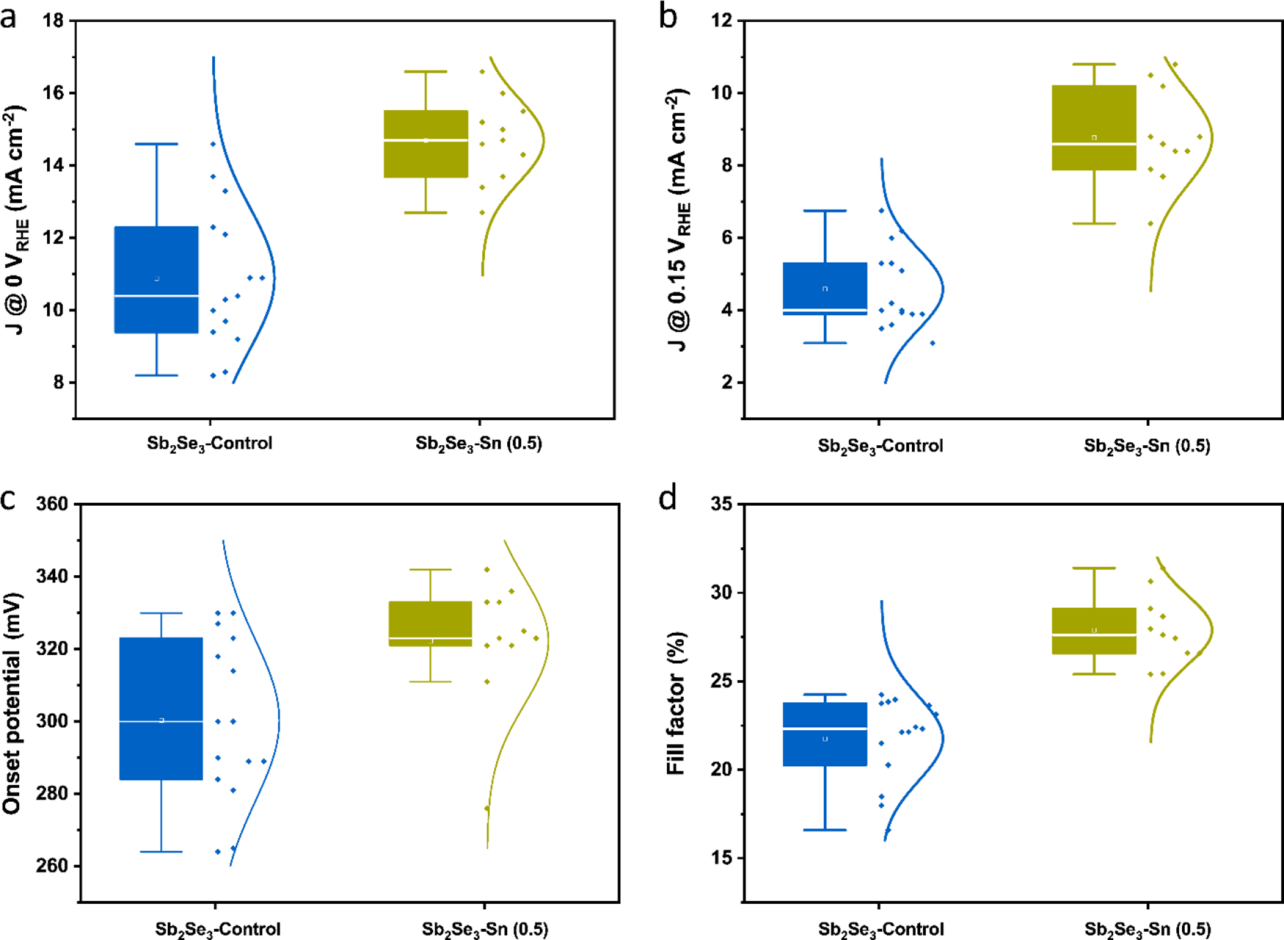
Statistical distribution based on more than ten devices for each condition: (a) photocurrent at 0 *V*_RHE_, (b) photocurrent at 0.15 *V*_RHE_, (c) onset potential, and (d) FF of Sb_2_Se_3_–Control and Sb_2_Se_3_–Sn (0.5) photocathodes.

Figure 2 shows the statistical distribution of *J*_ph_ at 0 *V*_RHE_, *J*_ph_ at 0.15 *V*_RHE_, onset potential (*V*_onset_), and FF for both Sb_2_Se_3_–Control and Sb_2_Se_3_–Sn (0.5) photocathodes. It is evident that the *J*_ph_ of Sb_2_Se_3_–Sn (0.5) is notably higher than that of Sb_2_Se_3_–Control photocathodes. The *V*_onset_ slightly increases from 300.2 ± 22.4 (Sb_2_Se_3_–Control) to 322.2 ± 17.6 mV for the Sb_2_Se_3_–Sn (0.5) photocathodes. In addition, the FF values display a rise from 21.7 ± 2.4% (Sb_2_Se_3_–Control) to 27.9 ± 1.9% (Sb_2_Se_3_–Sn (0.5)). It is unequivocal from these observations that the Sb_2_Se_3_–Sn (0.5) photocathodes outperform their Sb_2_Se_3_–Control counterparts. Furthermore, we performed the PEC stability test for the Sb_2_Se_3_–Sn (0.5) photocathode at a constant potential of 0.1 *V*_RHE_ under illumination (Figure S11). The *J*_ph_ of the Sb_2_Se_3_–Sn (0.5) photocathode steadily declined to 10.9 mA cm^–2^ after 1.7 h of illumination, which is approximately 77% of its initial value of 14.1 mA cm^–2^, which we attribute to the detachment of the Pt catalyst. After redepositing the Pt multiple times, the *J*_ph_ recovers its initial value and then gradually decreases again. As it is not the main focus of this work, we did not attempt to further improve the stability of the devices, which likely lose performance due to an unoptimized TiO_2_/cocatalyst interface.

The *J*_ph_ of a photocathode is governed by eq 1: *J*_abs_ is the light absorption rate expressed as the current density, η_sep_ is the charge separation efficiency of the photogenerated carriers, and η_inj_ is the charge injection efficiency at the interface between the electrode and the electrolyte for water reduction.^30^
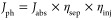
1Initially, the influence of η_inj_ can be disregarded, given the identical deposition of Pt cocatalyst on both Sb_2_Se_3_–Control and Sb_2_Se_3_–Sn (0.5) photocathodes.^23^ Thus, *J*_abs_ and η_sep_ emerge as the primary determinants of PEC performance. Direct measurement of *J*_abs_ for undoped and Sn-doped Sb_2_Se_3_ is challenging due to the opaque Mo substrate. Therefore, we combined ultraviolet–visible diffuse reflectance spectra (UV–vis DRS) and thickness data of Sb_2_Se_3_–Control and Sb_2_Se_3_–Sn (0.5) photocathodes to deduce the value of *J*_abs_. The UV–vis DRS spectrum of Sb_2_Se_3_–Control and Sb_2_Se_3_–Sn (0.5) photocathodes with the structure of Mo/Sb_2_Se_3_/TiO_2_ exhibits similar results, as seen in Figure S12. Based on the reflectance and identical thickness for Sb_2_Se_3_–Control and Sb_2_Se_3_–Sn (0.5) photocathodes (Figure S4), we can reasonably assume that their light absorption is roughly equal. The aforementioned analysis strongly suggests that the improvement in the PEC performance for the Sb_2_Se_3_–Sn (0.5) photocathode mainly originates from an increase in η_sep_.

### 2.3 Characterization of Sb_2_Se_3_

To elucidate the origin of the enhanced η_sep_ observed in Sb_2_Se_3_–Sn (0.5) photocathode, scanning electron microscopy (SEM) and X-ray diffraction (XRD) were first utilized to explore the morphology and orientation of undoped and Sn-doped Sb_2_Se_3_ films. Figure S13 displays the top-view and cross-sectional SEM images of undoped and Sn-doped Sb_2_Se_3_ films, clearly revealing the formation of compact, pinhole-free layers. From the cross-sectional SEM images, all Sb_2_Se_3_ films are stacked layer by layer, parallel to the Mo substrate. Observations from the high-magnification top-view SEM images (Figure S14) show small particles aggregating on the surfaces of the Sb_2_Se_3_–Sn (1.0) and Sb_2_Se_3_–Sn (1.5) films, which could be related to the noticeable decrease in PEC performance. To evaluate this assumption, the surface composition was examined using Raman spectra (see below).

As shown in Figure S15a, the crystal structures of undoped and Sn-doped Sb_2_Se_3_ films were examined by XRD. The observed diffraction peaks of all as-prepared Sb_2_Se_3_ films well matched with the standard PDF card (JCPDS 15-0861) of orthorhombic Sb_2_Se_3_, confirming the absence of secondary phase formation even at higher Sn^2+^ doping concentrations. Notably, the (020) peak shifts to a higher angle in the Sb_2_Se_3_–Sn (1.5) film, implying the incorporation of Sn^2+^ into the Sb_2_Se_3_ crystal lattice (Figure S15b). The evolution of crystal orientation in the Sb_2_Se_3_ films was evaluated by the texture coefficient (TC) according to the following equation^31^
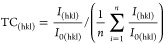
2where *I*_(hkl)_ and *I*_0(hkl)_ are the measured diffraction peak intensity and the corresponding peak intensity of the standard XRD pattern of Sb_2_Se_3_, respectively. The calculated TC values demonstrate that the as-prepared Sb_2_Se_3_ films are dominated by the (020) plane, and this effect becomes more prominent with increasing Sn doping concentrations (Figure S16). On the other hand, the intensity of the [hk1]-oriented planes shows a slight decrease. Previously reported hole mobilities of Sb_2_Se_3_ are 0.69, 1.17, and 2.59 cm^2^ V^–1^ s^–1^ in the [010], [100], and [001] directions, respectively.^32^ Therefore, we inferred that the crystal orientation evolution in the Sb_2_Se_3_–Sn (0.5) photocathode is not responsible for the PEC performance enhancement, primarily because of the lowest hole mobility in the [020] direction.

**Figure 3:**
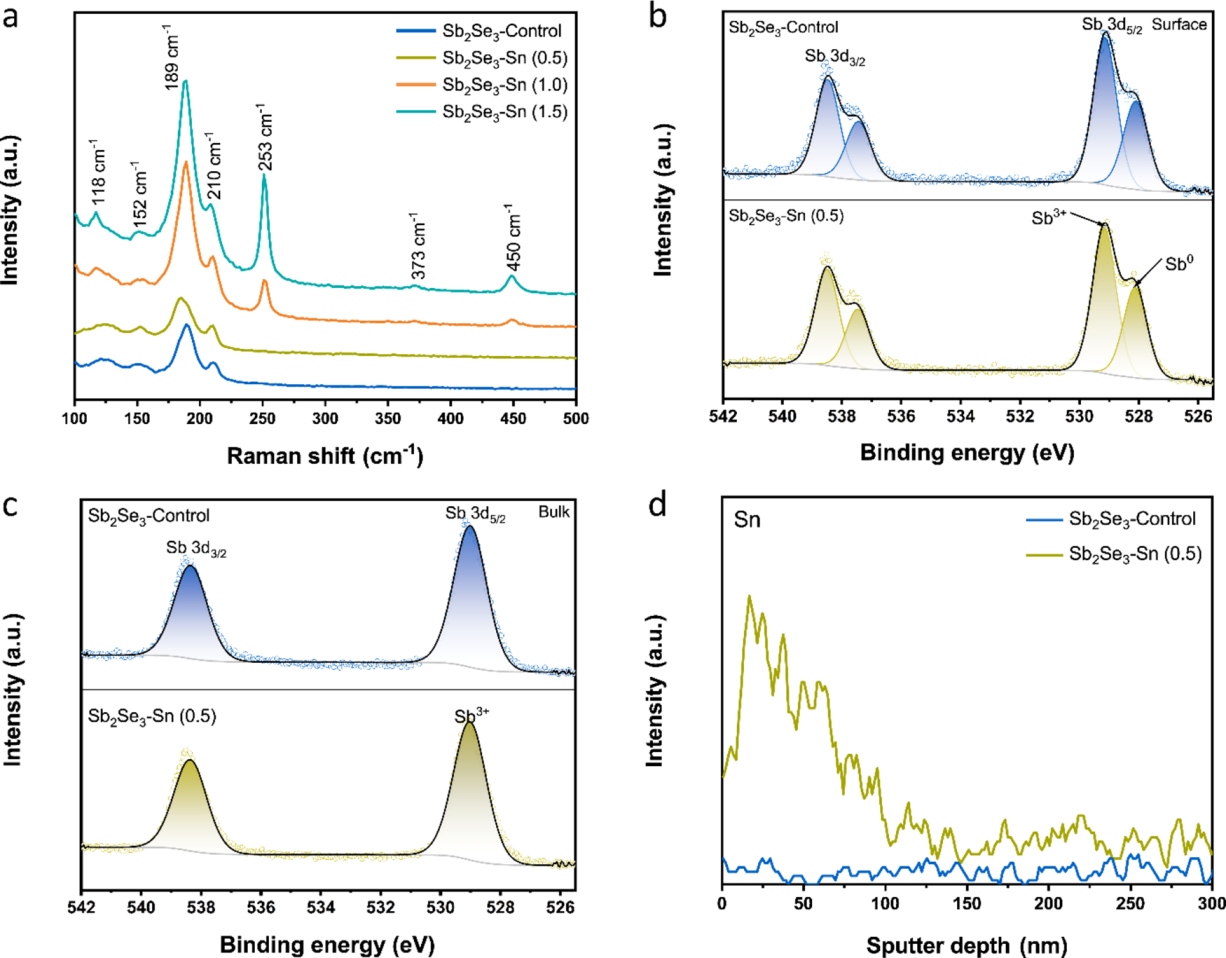
(a) Raman spectra of undoped and Sn-doped Sb_2_Se_3_ films. Sb 3d X-ray photoelectron spectroscopy (XPS) spectra of Sb_2_Se_3_–Control and Sb_2_Se_3_–Sn (0.5) films (b) before and (c) after sputter etching. (d) Time-of-flight secondary-ion mass spectrometry (ToF-SIMS) sputter depth profile of the Sb_2_Se_3_–Control and Sb_2_Se_3_–Sn (0.5) films.

Figure 3a displays the Raman spectra of undoped and Sn-doped Sb_2_Se_3_ films. All Sb_2_Se_3_ films show three typical peaks at 152, 189, and 210 cm^–1^, which are assigned to the orthorhombic Sb_2_Se_3_ phase.^33,34^ With a further increase in Sn doping concentrations, additional vibration modes at 118, 253, 373, and 450 cm^–1^ for Sb_2_Se_3_–Sn (1.0) and Sb_2_Se_3_–Sn (1.5) films are observed, suggesting the formation of Sb_2_O_3_.^12,27^ Considering these findings along with the SEM images (Figure S14), the presence of small particles on the surface of the Sb_2_Se_3_ films is assigned to Sb_2_O_3_. Based on these observations, we infer that the decreased PEC performance of the Sb_2_Se_3_–Sn (1.0) and Sb_2_Se_3_–Sn (1.5) photocathodes is attributed not only to the presence of Sb_2_O_3_, serving as recombination centers, but also to the increased intensity of the [020]-dominated crystal plane, reducing charge separation.^35^

X-ray photoelectron spectroscopy (XPS) characterization was carried out to characterize the chemical compositions of the undoped and Sn-doped Sb_2_Se_3_ films. Figures 3b and S17a show the Sb oxidation states on the surface of Sb_2_Se_3_ films. The typical XPS peaks at 529.1 and 538.5 eV confirm the formation of Sb_2_Se_3_, while peaks at 528.1 and 537.5 eV indicate the presence of Sb^0^ in undoped and Sn-doped Sb_2_Se_3_ films. It should be noted that no Sb_2_O_3_ was detected in the Sb_2_Se_3_–Control and Sb_2_Se_3_–Sn (0.5) films. In contrast, additional peaks located at 529.8 and 539.2 eV in the Sb_2_Se_3_–Sn (1.0) and Sb_2_Se_3_–Sn (1.5) films were attributed to Sb_5/2_ and Sb_3/2_ of Sb_2_O_3_, which also agrees with the Raman results. The absence of Sb_2_O_3_ in the Sb_2_Se_3_–Control and Sb_2_Se_3_–Sn (0.5) films suggests that the hydrothermal method significantly suppresses Sb_2_O_3_ formation, unlike physical deposition methods, where Sb_2_O_3_ commonly forms on the surface of Sb_2_Se_3_.^5,15,36^ After the removal of surface impurities by sputtering, only two peaks remained in undoped and Sn-doped Sb_2_Se_3_ films, indicating the phase purity in the bulk of the Sb_2_Se_3_ films (Figures 3c and S17b). The binding energies at 54.0 and 54.8 eV are ascribed to Se 3d_5/2_ and Se 3d_3/2_ of Sb_2_Se_3_, as shown in Figure S18. The presence of Sn was observed in the Sb_2_Se_3_–Sn (1.5) film, as seen in Figure S17c,d. The binding energies of Sn 3d_5/2_ and Sn 3d_3/2_ are found to be 485.6 and 494.0 eV, which is consistent with a chemical state of Sn^2+^ in the Sb_2_Se_3_–Sn (1.5) film. However, XPS is not sensitive enough to detect the presence of the Sn element in the Sb_2_Se_3_–Sn (0.5) film due to its low concentration. To gain insight into the distribution of the Sn element, time-of-flight secondary ion mass spectrometry (ToF-SIMS) was conducted on Sb_2_Se_3_–Control and Sb_2_Se_3_–Sn (0.5) films. The ToF-SIMS results in Figure 3d indicate that no Sn was detected in the Sb_2_Se_3_–Control film. In contrast, the signal of Sn gradually declines and then becomes uniformly distributed with probing depth in the Sb_2_Se_3_–Sn (0.5) films, suggesting the accumulation of Sn near the surface of Sb_2_Se_3_. Figure S19 reveals that Sb and Se are uniformly distributed in both undoped and Sn-doped Sb_2_Se_3_ films.

### 2.4 Carrier Density and Open-Circuit Potential (OCP) Measurements

**Figure 4:**
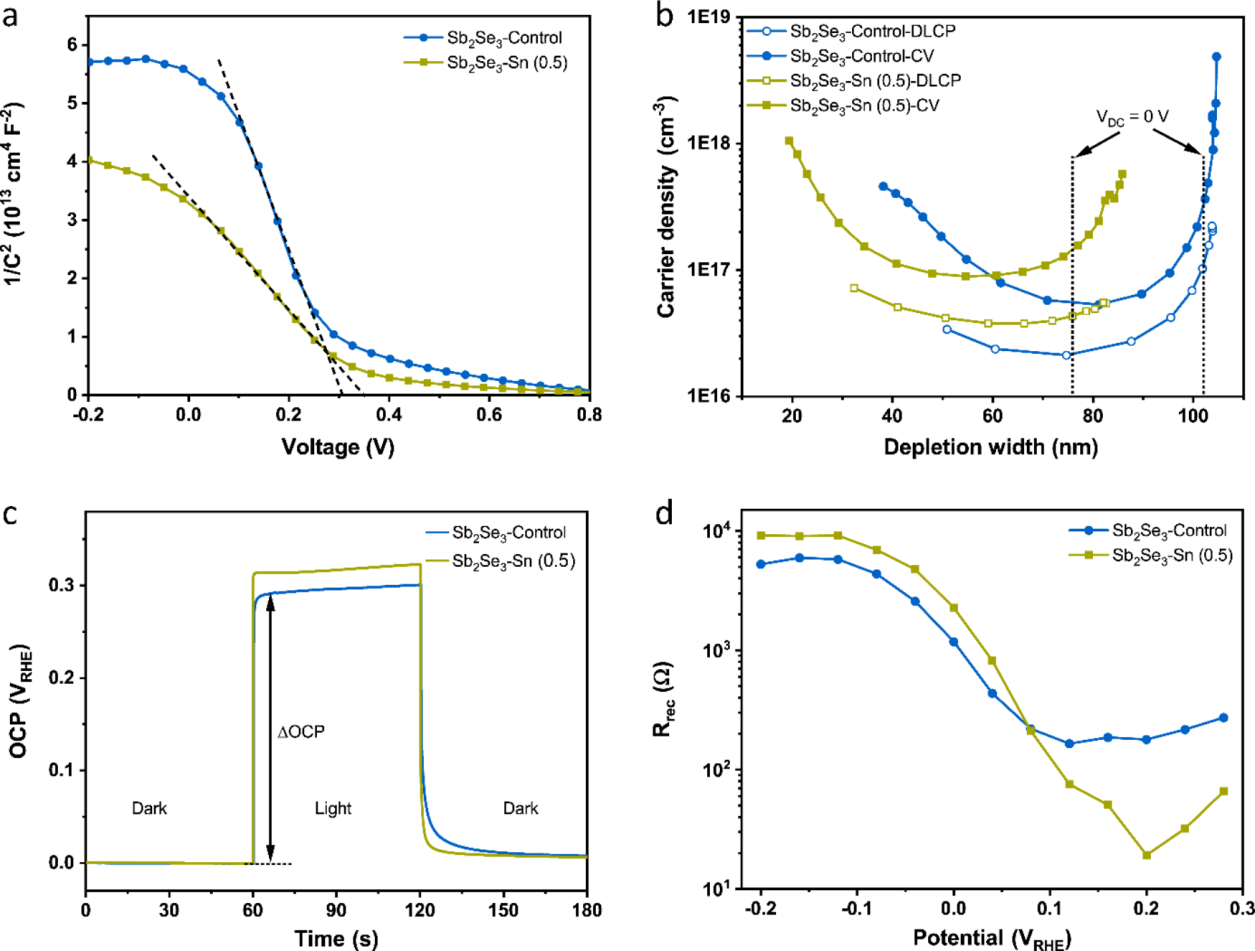
(a) Mott–Schottky plots, (b) CV and DLCP profiles, (c) OCP versus time under off/on cycles of light illumination, and (d) *R*_rec_ of Sb_2_Se_3_–Control and Sb_2_Se_3_–Sn (0.5) photocathodes obtained from electrochemical impedance spectroscopy (EIS) ((a, b) in 2-electrode mode and (c, d) in 3-electrode mode).

We further performed capacitance–voltage (CV) and drive-level capacitance profiling (DLCP) measurements to characterize the carrier density in the Sb_2_Se_3_–Control and Sb_2_Se_3_–Sn (0.5) devices.^37^ The measurements were performed utilizing the device structure of Mo/Sb_2_Se_3_/TiO_2_/Al employing a two-electrode system under dark conditions (Figure S20). From the Mott–Schottky plots (Figures 4a and S21), the flat band voltage (*V*_FB_) can be extracted by linear fitting and extrapolation to the intercept with the *x*-axis. The *V*_FB_ values of the Sb_2_Se_3_–Control devices are in the range of 0.30–0.42 V, whereas the *V*_FB_ of the Sb_2_Se_3_–Sn (0.5) devices remains in a narrow range of 0.34–0.38 V. This spread of *V*_FB_ values agrees well with the *V*_onset_ results observed in Figure 2c, where the *V*_onset_ of the Sb_2_Se_3_–Control photocathode is distributed over a larger range, while the *V*_onset_ of the Sb_2_Se_3_–Sn (0.5) photocathode has less variation. The minimal discrepancy between *V*_onset_ and *V*_FB_ in the Sb_2_Se_3_–Sn (0.5) is indicative of reduced surface defects with the Sn^2+^ treatment (see below). In general, the carrier density of CV profiling (*N*_CV_) includes the response of free carriers, bulk defects, and surface defects, while the carrier density obtained from DLCP profiling measurements (*N*_DLCP_) is insensitive to surface defects.^38^ As seen in Figure 4b, U-shaped profiles of *N*_CV_ and *N*_DLCP_ were observed in both Sb_2_Se_3_ devices, which arises from the punch-through effect due to the relatively thin nature of the Sb_2_Se_3_ films (around 270 nm thickness, see Supporting Note 1 for further details).^39^ We therefore assigned the carrier density based on the lowest point of the DLCP profiles. As mentioned above, an increase in the p-type conductivity of Sb_2_Se_3_ with Sn doping treatment has been reported elsewhere.^20^ This phenomenon was also found in our Sb_2_Se_3_ samples. The carrier density of the Sb_2_Se_3_–Sn (0.5) sample is 3.77 × 10^16^ cm^–3^, which is almost twice as high as that of the Sb_2_Se_3_–Control sample (2.12 × 10^16^ cm^–3^). Considering the higher doping density of the ALD TiO_2_ (2.6 × 10^20^ cm^–3^),^40^ the depletion layer is predominantly located within the Sb_2_Se_3_ layer near the Sb_2_Se_3_/TiO_2_ junction. The surface defect density was calculated by subtracting *N*_DLCP_ from *N*_CV_ at 0 V and multiplying this value by the depletion layer width (*W*_d_).^22,41^ The corresponding surface defect density for Sb_2_Se_3_–Control and Sb_2_Se_3_–Sn (0.5) devices were calculated to be 2.67 × 10^12^ and 8.64 × 10^11^ cm^–2^, respectively. The obtained parameters are summarized in Table S1. We assume that the suppressed formation of surface defects could be attributed to the accumulation of Sn near the surface of the Sb_2_Se_3_–Sn (0.5) photocathode. The increased carrier density and reduced surface defects play a crucial role in the PEC performance enhancement of the Sb_2_Se_3_–Sn (0.5) photocathode through reduced interface recombination and better charge collection. Compared to the Sb_2_Se_3_–Control photocathode with a W_d_ of 102 nm, the calculated W_d_ of the Sb_2_Se_3_–Sn (0.5) photocathode drops to 76 nm due to the increased carrier density.

To assess the thermodynamic driving force for charge separation, we performed an open-circuit potential (OCP) test.^42,43^ The OCP measurement was conducted in a black box under H_2_ purging (details in the Experimental Section). The changes in OCP between dark (OCP_dark_) and illuminated conditions (OCP_light_) for undoped and Sn-doped Sb_2_Se_3_ photocathodes are illustrated in Figure 4c. It is noteworthy that the Fermi levels of both photocathodes equilibrate with the redox potential (*E*_H/H_2__^+^, 0 *V*_RHE_) in the dark condition. Under illumination, the quasi-Fermi level of holes will shift to a more positive potential. The difference between OCP_dark_ and OCP_light_ is the photovoltage (*V*_ph_). A larger *V*_ph_ of 0.32 *V*_RHE_ was observed for the Sb_2_Se_3_–Sn (0.5) photocathode, demonstrating improved charge separation and an earlier onset potential in the water splitting experiments. Another way to evaluate the quality of Sb_2_Se_3_ is to study the photogenerated carrier lifetime as a function of OCP decay. Figure S22 displays the normalized transient OCP decay after stopping illumination (see Supporting Note 2 for further details).

### 2.5 EIS Measurement

Electrochemical impedance spectroscopy (EIS) was conducted to further reveal the efficient charge separation kinetics within the Sb_2_Se_3_ photocathodes. EIS measurements were recorded under 10% light illumination with an applied potential ranging from 0.4 to −0.2 *V*_RHE_. To ensure device stability, cyclic voltammetry measurements were recorded before and after the EIS tests, as shown in Figure S23. Figure S24 shows the Nyquist plots for Sb_2_Se_3_–Control and Sb_2_Se_3_–Sn (0.5) photocathodes, and an equivalent circuit was used to fit the Nyquist curves. Here, the parameter of charge recombination resistance (*R*_rec_) represented by the semicircle in the Nyquist plots at the lowest frequencies is the prime concern. As seen from Figure S24, the semicircle diameter for the Sb_2_Se_3_–Sn (0.5) photocathode is larger than that of the Sb_2_Se_3_–Control photocathode, implying a higher charge separation efficiency in the former. The extracted *R*_rec_ values for both Sb_2_Se_3_ photocathodes from the EIS equivalent circuit fitting are presented in Figure 4d. The *R*_rec_ of the Sb_2_Se_3_–Sn (0.5) photocathode shows a sharp and early rise compared to the Sb_2_Se_3_–Control photocathode. The *R*_rec_ of the Sb_2_Se_3_–Sn (0.5) photocathode is approximately two times higher compared to the Sb_2_Se_3_–Control photocathode at potentials below 0.08 *V*_RHE_. This is indicative that the incorporation of Sn^2+^ into Sb_2_Se_3_ suppresses charge recombination. This increased *R*_rec_ value for Sn-doped Sb_2_Se_3_ is in good agreement with the OCP enhancement, which promotes charge separation. Therefore, with the optimal concentration of Sn doping, the Sb_2_Se_3_ films effectively increase *R*_rec_ and therefore reduce recombination within the semiconductor, resulting in improved PEC performance.

## 3 Conclusions

In summary, we have developed a simple and effective hydrothermal method to synthesize high-quality Sb_2_Se_3_ films, effectively reducing surface defects by incorporating Sn^2+^ as a dopant. Moreover, the p-type conductivity is enhanced in Sn-doped Sb_2_Se_3_. OCP and EIS tests confirmed improved charge separation efficiency in the Sb_2_Se_3_–Sn (0.5) photocathode compared to the Sb_2_Se_3_–Control device. The photocurrent density increased from 14.4 mA cm^–2^ (Sb_2_Se_3_–Control) to 17.1 mA cm^–2^ (Sb_2_Se_3_–Sn (0.5)) at 0 *V*_RHE_, with FF rising from 21.5 to 28.7%. These findings underscore the potential of our approach to enhance Sb_2_Se_3_ photocathode performance in PEC applications. Future efforts should focus on optimizing film thickness and orientation, aiming for improved light absorption and enhanced charge separation efficiency, thus further enhancing PEC performance.

## 4 Experimental Section

### 4.1 Hydrothermal Deposition of Sb_2_Se_3_ Films

All Sb_2_Se_3_ films were deposited onto molybdenum (Mo)-coated glass substrates (Guluo, Luoyang). Before deposition, the Mo-coated glass was cleaned with acetone, soapy water, Milli-Q water, and isopropanol in an ultrasonic bath for 10 min each. After cleaning, the Mo-coated glass substrates were dried under N_2_ flow. The Sb_2_Se_3_ films were fabricated via a hydrothermal method, utilizing potassium antimony tartrate trihydrate (Sigma-Aldrich, ≥99%) and selenourea (Sigma-Aldrich, 98%) as the Sb and Se sources, respectively. First, 0.334 g (10 mM) of K_2_Sb_2_(C_4_H_2_O_6_)_2_·3H_2_O and 0.126 g (20 mM) of Na_2_SO_3_ (Sigma-Aldrich, ≥98%) were sequentially added to a beaker containing 50 mL of Milli-Q water (18.2 MΩ·cm). The solution was stirred at 400 rpm for 5 min after each addition. The addition of Na_2_SO_3_ caused an increase in the pH of the solution (from pH of 4.13 to pH of 6.89), leading to the formation of Sb_2_O_3_.
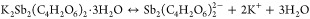
3
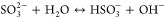
4
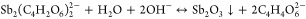
5Subsequently, 0.246 g (40 mM) of (NH_2_)_2_CSe was added to the above solution, leading to a decrease in pH to 6.61
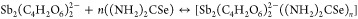
6
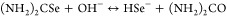
7To remove the Sb_2_O_3_ precipitation, the as-prepared solution was allowed to precipitate for 4 h and then filtered with filter paper. For the fabrication of Sn-doped Sb_2_Se_3_ films, various amounts of SnSO_4_ (0.1, 0.2, and 0.3 mM) as a dopant were added to the filtered solution. The resulting solution was then transferred into a 100 mL Teflon-lined hydrothermal reactor. Mo-coated glass substrates, partially wrapped with Teflon tape and placed face down in a holder, were positioned in the Teflon tank. The reaction was held at 165 °C for 4 h in an oven. Following the reaction, the autoclave was naturally cooled to room temperature. Then, the Sb_2_Se_3_ films were successfully deposited on the Mo substrates.
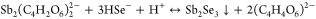
8The Sb_2_Se_3_ films were then rinsed with deionized water and dried with flowing N_2_ in ambient air.

### 4.2 Deposition of TiO_2_ Layer and Pt Catalyst

A 30 nm layer of TiO_2_ was deposited onto the Sb_2_Se_3_ films using a thermal atomic layer deposition (ALD) system (PICOSUN, R200). The tetrakis(dimethylamino)titanium (TDMAT, Aldrich, 99.99%) and Milli-Q H_2_O were employed as the precursors for Ti and O sources, respectively. During deposition, the reaction chamber temperature was kept at 120 °C, while the temperature for the TDMAT was maintained at 85 °C. Pt was used as the hydrogen evolution reaction catalyst. Nominally 2 nm of Pt was sputtered onto the surface of the TiO_2_ using a sputter coater (LEICA EM ACE600). The thickness of the deposited Pt was monitored by a gold-coated quartz microbalance.

### 4.3 PEC Performance and EIS Measurements

For PEC performance measurements, a three-electrode electrochemical cell with a potentiostat (BioLogic SP-300) was used. The light source consisting of AM 1.5 G filter was provided by a 150 W Xe-lamp to simulate AM 1.5 G sunlight. The light intensity was calibrated using a silicon diode from PV Measurements, Inc. (100 mW cm^–2^). A Pt wire and an Ag/AgCl electrode (KOSLOW, saturated KCl, +0.197 V vs normal hydrogen electrode) were used as the counter and reference electrodes, respectively. The electrolyte used for the measurements was 1 M H_2_SO_4_ (pH = 0) electrolyte. The *J*–*V* curves were recorded under intermittent or continuous light illumination with a scan rate of 10 mV s^–1^. IPCE measurements were performed using a home-built system equipped with a halogen light source and a double monochromator. Before the IPCE measurements, the light intensity was calibrated with a silicon photodiode. EIS measurement was scanned from 0.4 to −0.2 V with a small potential amplitude of 15 mV under 10% light illumination, applying an AC perturbation frequency ranging from 7 MHz to 0.2 Hz. The Nernst equation was applied to convert the potentials (vs Ag/AgCl) into the reversible hydrogen electrode (RHE) scale.
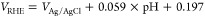
9

### 4.4 OCP and Light Intensity Dependence of Photocurrent Measurements

For the OCP measurement in 1 M H_2_SO_4_, a potentiostat with two channels was used in a black box. One channel was used in a typical three-electrode system with a Ag/AgCl reference electrode, a Pt wire counter electrode, and the Sb_2_Se_3_ photocathode as the working electrode. The second channel was used in a 2-electrode configuration between the same counter electrode and an additional platinum wire that was placed just underneath the working electrode. Prior to the OCP measurement (using the first channel), electrolysis was carried out using the second channel for 30 min, which ensured saturation of the electrolyte solution with H_2_ in the vicinity of the Sb_2_Se_3_ photocathode surface (Figure 4c indeed shows that the potential of the Sb_2_Se_3_, calculated according to eq 10, is 0 *V*_RHE_ in the dark). The light intensity dependence of the photocurrent measurement was performed at 0 *V*_RHE_ using a three-electrode system. For both measurements, an array of nine white light diodes was used (SP-12-W5, cool white Luxeon Rebel) controlled by a Keithley power source (PSW4323).

### 4.5 Material Characterization

A Zeiss Gemini 450 SEM was used to determine the morphology of the undoped and Sn-doped Sb_2_Se_3_ samples. The crystalline phase of the obtained Sb_2_Se_3_ films was detected by XRD (Rigaku Smartlab) with Cu Kα radiation (λ = 0.15406). The white powder obtained from the solution precipitation was examined by PXRD. The reflectance spectra of the Sb_2_Se_3_/TiO_2_ photocathodes were characterized using UV–vis DRS recorded on a Shimadzu UV-3600 spectrometer. Raman spectra were measured on a Renishaw inVia Raman microscope with a 532 nm laser. XPS measurements were performed using a physical electronics Quantum 2000 X-ray photoelectron spectrometer featuring monochromatic Al Kα radiation, operated at 15 kV and 32.3 W. The energy scale of the instrument was calibrated by an Au reference sample. The analysis was conducted at a vacuum level of 1 × 10^–6^ Pa, employing an electron takeoff angle of 45° and a pass energy of 23.5 eV. Instrument-specific sensitivity factors were applied for Shirley background subtraction, and core-level spectra were plotted to deconvolute spectra with contributions from multiple elements as necessary. A GL 30 asymmetric line shape was assumed for the core-level emissions, with a Δ*E* of 9.34 eV for the Sb 3d doublet and a Δ*E* of 0.86 eV for the Se 3d.

### 4.6 ToF-SIMS Measurements

Secondary-ion mass spectrometry measurements were carried out using a ToF-SIMS 5 instrument from IONTOF GmbH, Germany. The instrument operated in depth profiling mode, performing simultaneous dual-beam sputtering and analysis. Bi^+^ primary ions with current of 1.5 pA were employed for the elemental analysis in positive polarity of the undoped and Sn-doped Sb_2_Se_3_ samples. Sputtering was conducted with O_2_ ions with energy of 1000 eV and sputtering current of 287 nA. The sputtering ion beam was rastered over a 500 μm × 500 μm area, with the primary beam overlaid in the center of the sputter area and rastered over 200 μm × 200 μm area. The sputter rate is around 1.3 nm/s.

### 4.7 CV and DLCP Measurements

To perform CV and DLCP measurements, 100 nm of TiO_2_ and Al layers were deposited onto the Sb_2_Se_3_ surface, respectively. Standard CV and DLCP measurements were conducted using a potentiostat (Biologic SP-200) in a two-electrode configuration under dark condition at room temperature. For the standard CV measurements, a DC bias was scanned from −0.5 to 1 V with an AC amplitude of 20 mV. The DLCP measurements were conducted with an AC amplitude ranging from 20 to 120 mV and a DC bias range of −0.2 to 0.5 V. For each AC bias, an additional offset DC voltage was applied to maintain a constant maximum forward bias. An AC frequency of 10 kHz was applied to extract the carrier density in both CV and DLCP measurements. The carrier density from the CV measurement (*N*_CV_) can be extracted by the following equation
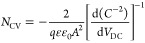
10Here, *q* represents the elementary charge, ε represents the dielectric constant of the Sb_2_Se_3_ (taken as 15.1),^44^ ε_0_ is the permittivity of free space, and *A* is the measured area of the diode. In the DLCP measurements, the carrier density is derived from the nonlinear relationship between the change in charges (δ*Q*) and the perturbation AC bias (δ*V*)
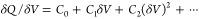
11The values of *C*_0_ and *C*_1_ can be obtained by fitting the capacitance versus perturbation AC bias plots with this function. The carrier density from the DLCP measurement (*N*_DLCP_) can be calculated by
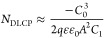
12The profiling depth from the barrier junction < χ > for the CV (taking *C* from eq 10) and DLCP (taking *C*_0_ from eq 12) can be determined by
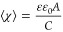
13More details can be found in our previous paper.^45^

## Supporting Information Available

Preparation of Sb_2_Se_3_ films; XRD pattern; SEM and EDS mapping images; reflectance spectra; XPS measurements; PEC performance; ToF-SIMS measurements; OCP and EIS measurements; device structure for CV and DLCP test; and additional discussions including OCP decay, CV, and DLCP (PDF)
