# Supplementary material for: One-Step Hydrothermal Synthesis of Sn-Doped Sb2Se3 for Solar Hydrogen Production
Source: ACS Catal. 2024 Jun 18;14(13):9877–86. doi: 10.1021/acscatal.4c01762 (PMC11232013; doi:10.1021/acscatal.4c01762)
Supplement: Supplementary file 1 — cs4c01762_si_001.pdf [file cs4c01762_si_001.pdf]

Supporting Information

One-step Hydrothermal Synthesis of Sn-doped Sb_2Se_3 for Solar Hydrogen Production

*Zhenbin Wang[a], Sanghyun Bae[a], Miloš Baljozović[b], Pardis Adams[a], David Yong[a], Erin Service[a], Thomas Moehl[a], Wenzhe Niu[a,c], S. David Tilley[a]**

[a] Department of Chemistry, University of Zurich, Winterthurerstrasse 190, 8057 Zurich, Switzerland.

[b] Molecular Surface Science Group, Empa, Swiss Federal Laboratories for Materials Science and Technology, 8600 Dübendorf, Switzerland

[c] Laboratory of Photonics and Interfaces, Institute of Chemical Sciences and Engineering, École Polytechnique Fédérale de Lausanne, Lausanne 1015, Switzerland

*Email: david.tilley@chem.uzh.ch

S1. Precursor solution preparation process....................3
S2. XRD of precipitation....................3
S3. Sb_2Se_3 thin film preparation process....................3
S4. The thickness of Sb_2Se_3 films....................4
S5. The Sb_2Se_3 film without Na_2SO_3....................4
S6. EDS of Sb_2Se_3 device....................5
S7-8. PEC performance of undoped and Sn-doped Sb_2Se_3 photocathodes....................6
S9. Light intensity dependence....................7
S10. IPCE of Sb_2Se_3 photocathodes....................7
S11. Stability of Sb_2Se_3-Sn (0.5) photocathode....................8
S12. The reflectance of undoped and Sn-doped Sb_2Se_3 devices....................8
S13-14. SEM images of Sb_2Se_3 films....................9
S15-16. XRD patterns of undoped and Sn-doped Sb_2Se_3 films....................10
S17-18. XPS spectra of Sb_2Se_3 films....................11
S19. ToF-SIMS of for Sb_2Se_3 films....................12
S20. Device configuration for CV and DLCP measurements....................13
S21. Mott-Schottky plots....................13
S22. OCP decay....................14
S23. Cyclic voltammetry before and after EIS measurements....................14
S24. Nyquist plots....................15
Supplementary Note 1....................15
Supplementary Note 2....................16
References....................17

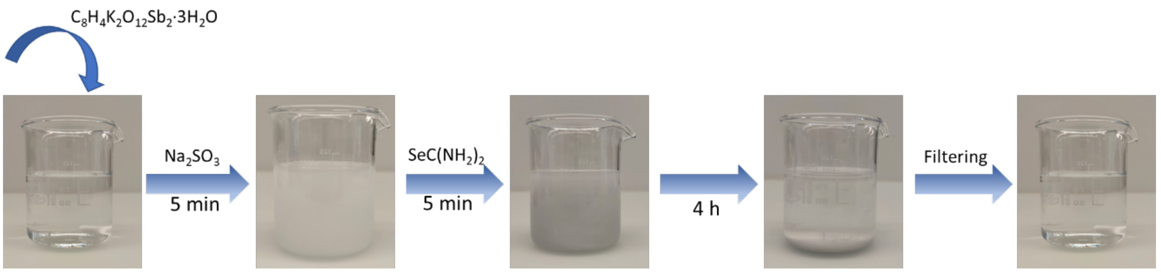

Figure S1. Schematic illustration of the solution preparation process. $SnSO_4$ was added as a dopant after the filtration process.

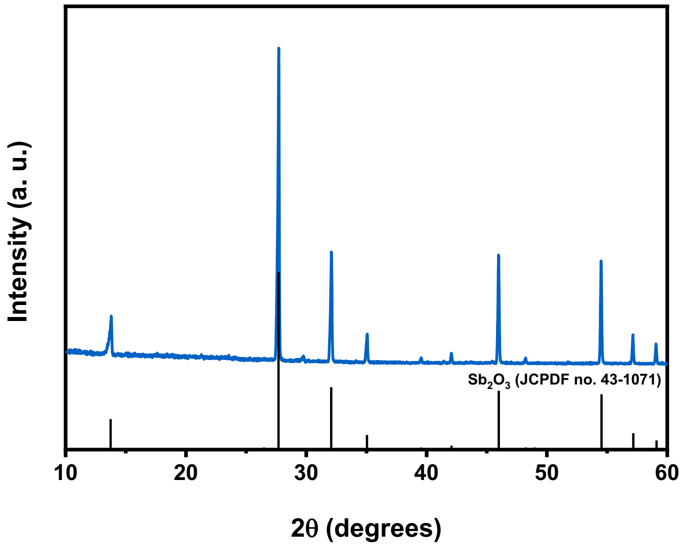

Figure S2. XRD pattern of the Sb_2O_3 powders obtained from the solution precipitation. All peaks correspond to Sb_2O_3 except for the peak at 29 degrees, which corresponds to elemental selenium.

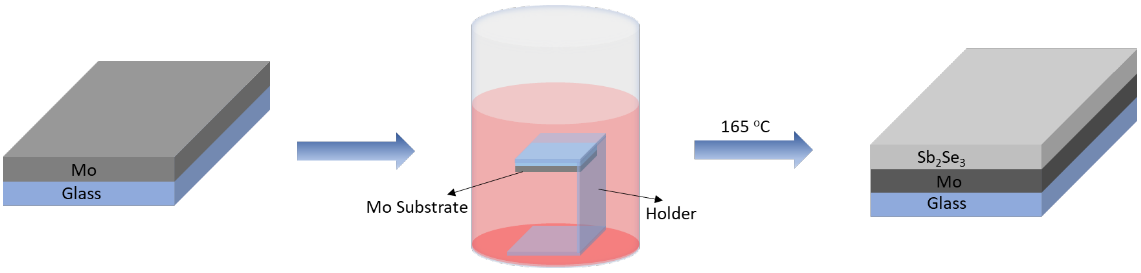

Figure S3. Schematic illustration of the Sb_2Se_3 thin film fabrication process. Mo-coated glass substrate held by a Teflon holder, facing down towards the solution.

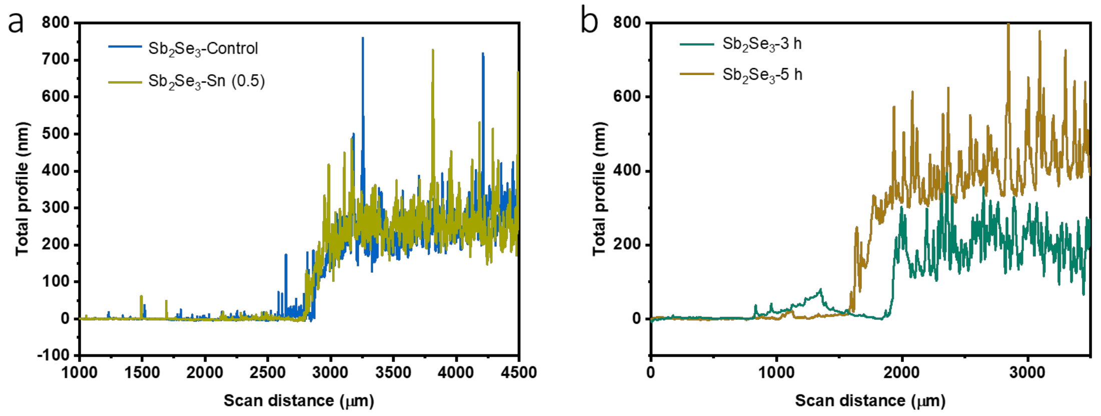

Figure S4. (a) The thickness of Sb_2Se_3-Control, Sb_2Se_3-Sn (0.5) prepared at 165 °C for 4 h. (b) The thickness of Sb_2Se_3-3h, and Sb_2Se_3-5h films prepared at 165 °C for 3 h and 5 h.

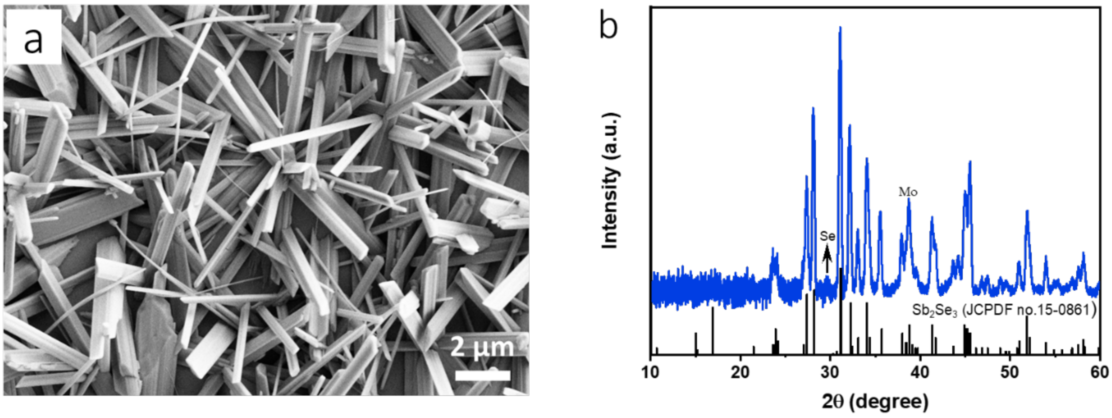

Figure S5. (a) The SEM image of the Sb_2Se_3 film that was prepared without the addition of Na_2SO_3. (b) XRD pattern of the corresponding Sb_2Se_3 film. All peaks correspond to Sb_2Se_3 except for the peak at 29 degrees, which corresponds to elemental selenium.

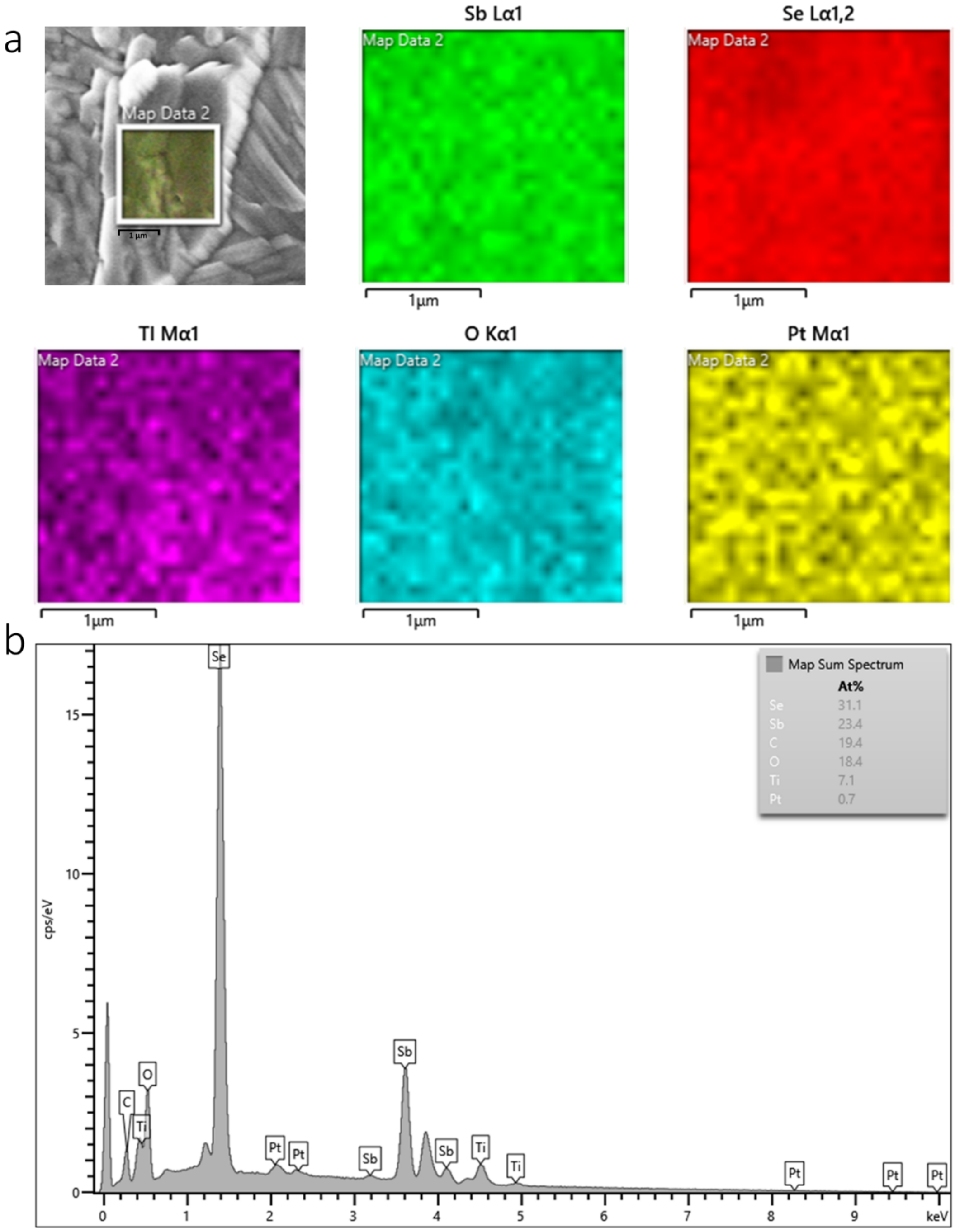

Figure S6. (a) EDS mapping and (b) the corresponding EDS spectra of the Sb_2Se_3 thin film covered with TiO_2 and Pt.

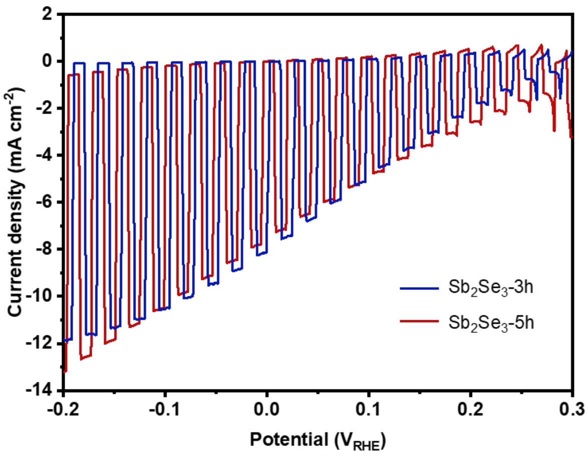

Figure S7. LSV plots of the Sb_2Se_3-3h and Sb_2Se_3-5h photocathodes.

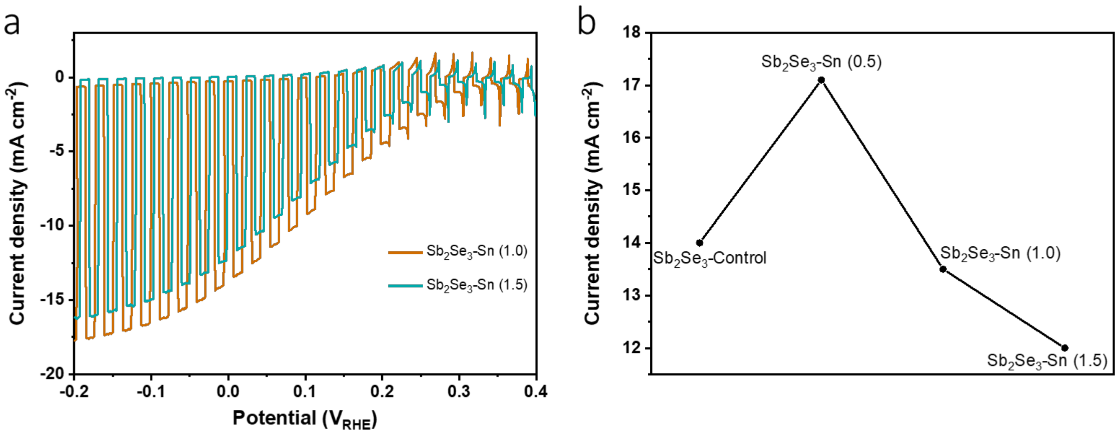

Figure S8. (a) LSV plots of Sb_2Se_3-Sn (1.0) and Sb_2Se_3-Sn (1.5) photocathodes. (b) Photocurrent density at 0 V_{RHE} of the best devices for undoped and Sn-doped Sb_2Se_3.

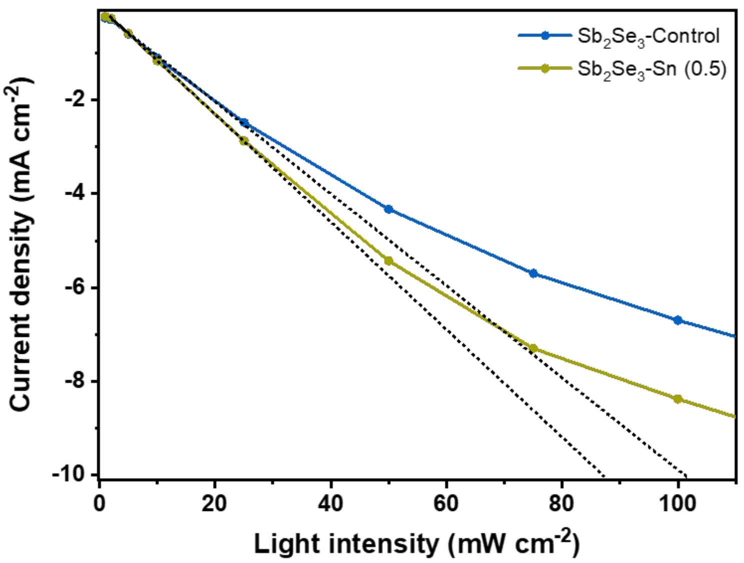

Figure S9. Dependence of photocurrent on white light intensity at 0 V_{RHE} for Sb_2Se_3-Control and Sb_2Se_3-Sn (0.5) photocathodes.

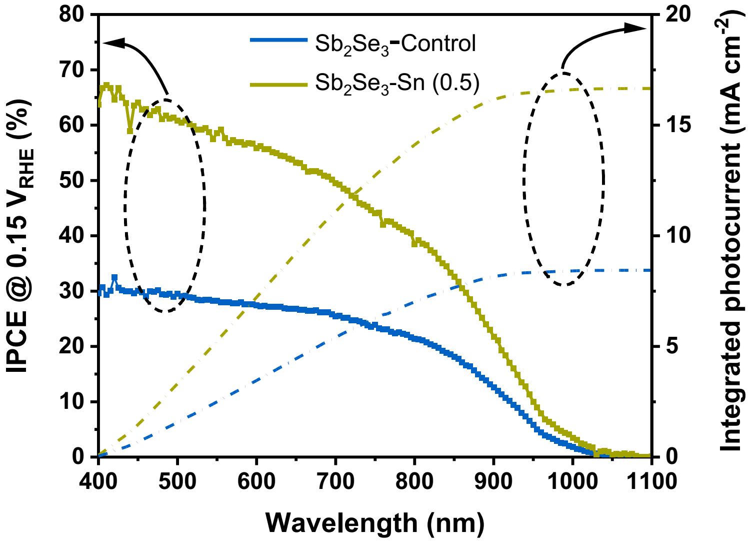

Figure S10. IPCE and integrated photocurrent (dashed line) of Sb_2Se_3-Control and (b) Sb_2Se_3-Sn (0.5) photocathodes at 0.15 V_{RHE} under 10% white light bias.

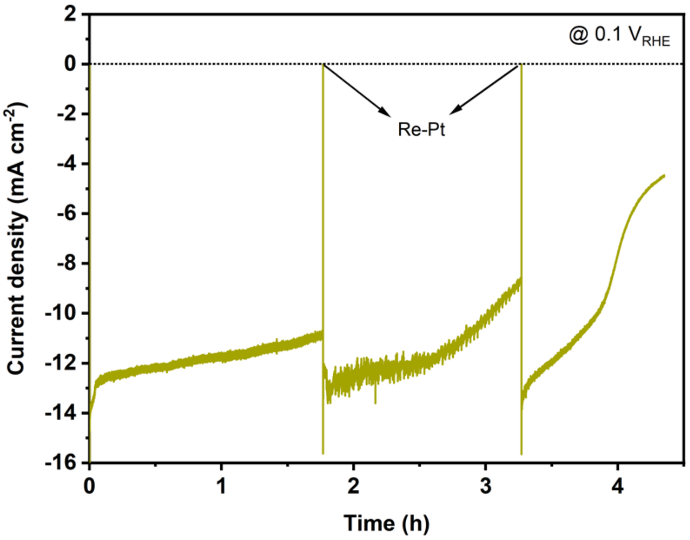

Figure S11. Stability tests of the Sb_2Se_3-Sn (0.5) photocathode under simulated solar light illumination (AM 1.5 G, 100 mW cm^{-2}) were recorded at 0.1 V_{RHE} in 1 M H_2SO_4 solution (pH = 0).

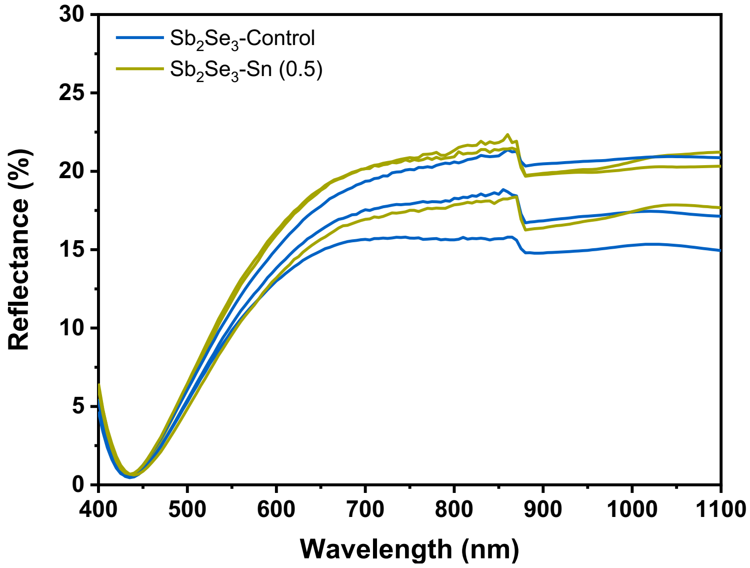

Figure S12. Diffuse reflectance spectra of Sb_2Se_3-Control and (b) Sb_2Se_3-Sn (0.5) films with the structure of Mo/Sb_2Se_3/TiO_2. Three films for each were characterized.

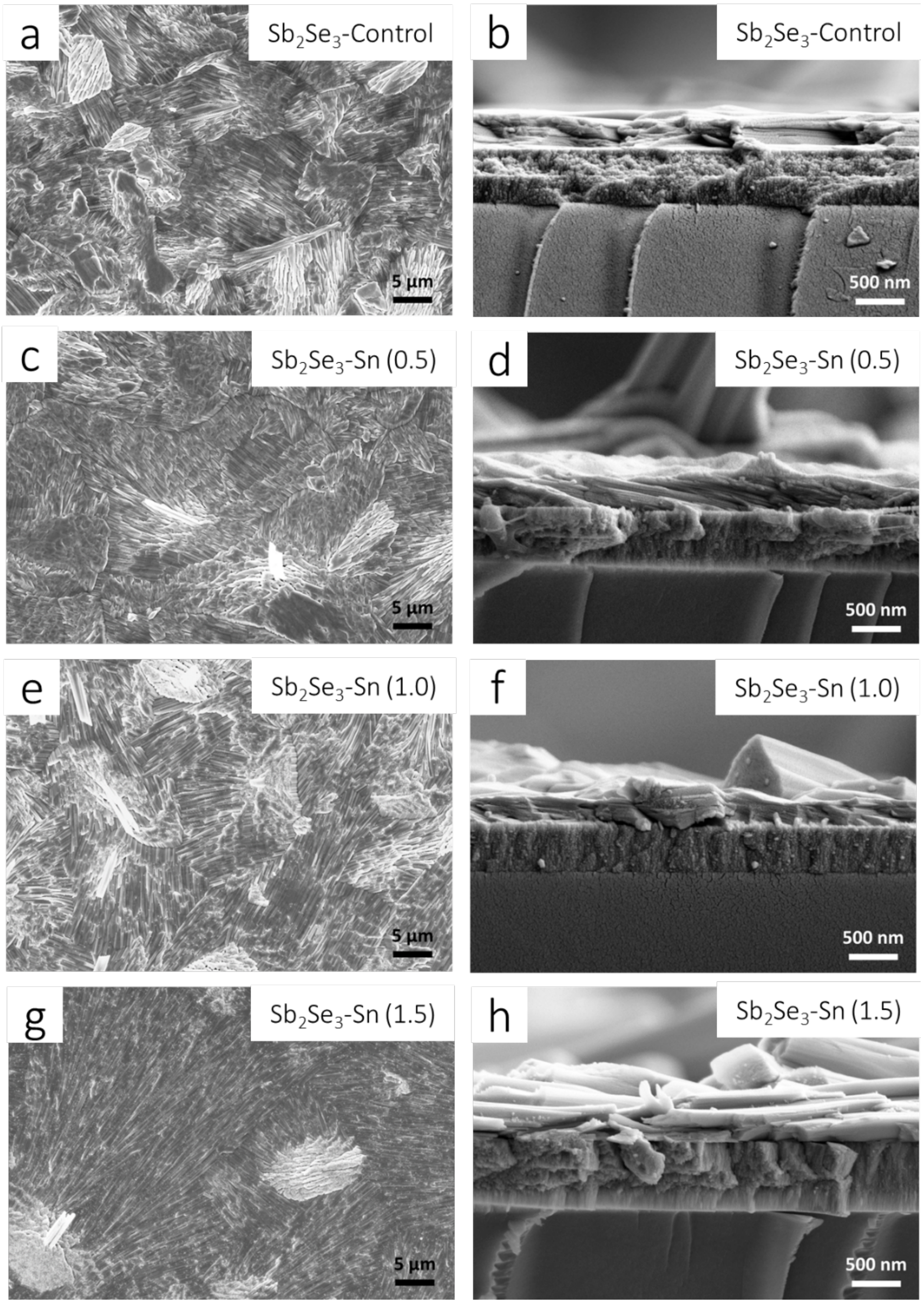

Figure S13. Top-view and the corresponding cross-sectional SEM images of (a, b) Sb_2Se_3-Control, (c, d) Sb_2Se_3-Sn (0.5), (e, f) Sb_2Se_3-Sn (1.0), and (g, h) Sb_2Se_3-Sn (1.5) films.

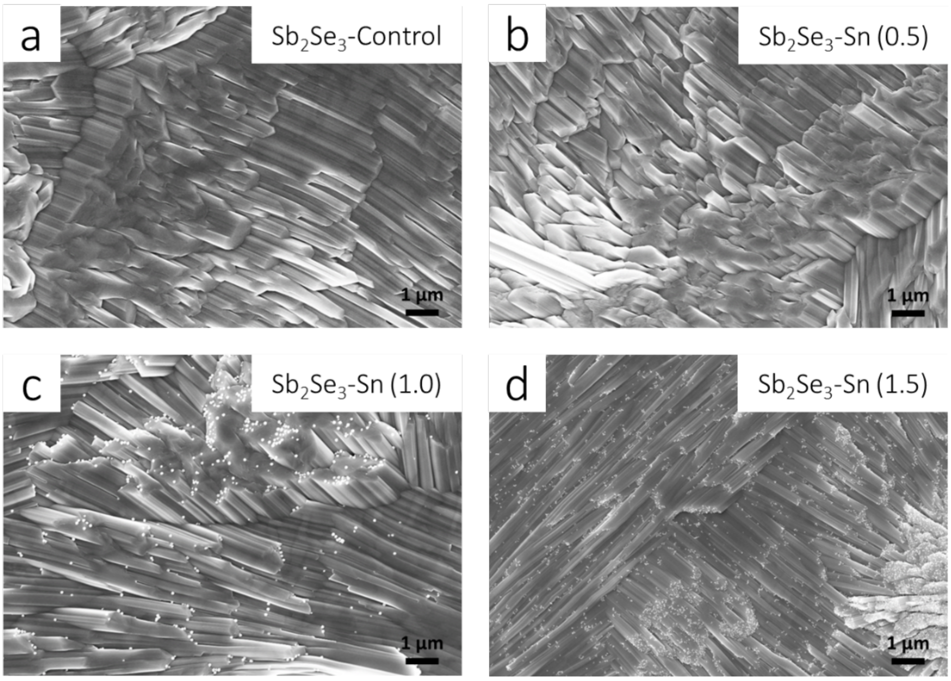

Figure S14. High magnification top-view SEM images of (a) Sb_2Se_3-Control, (b) Sb_2Se_3-Sn (0.5), (c) Sb_2Se_3-Sn (1.0), and (d) Sb_2Se_3-Sn (1.5) films.

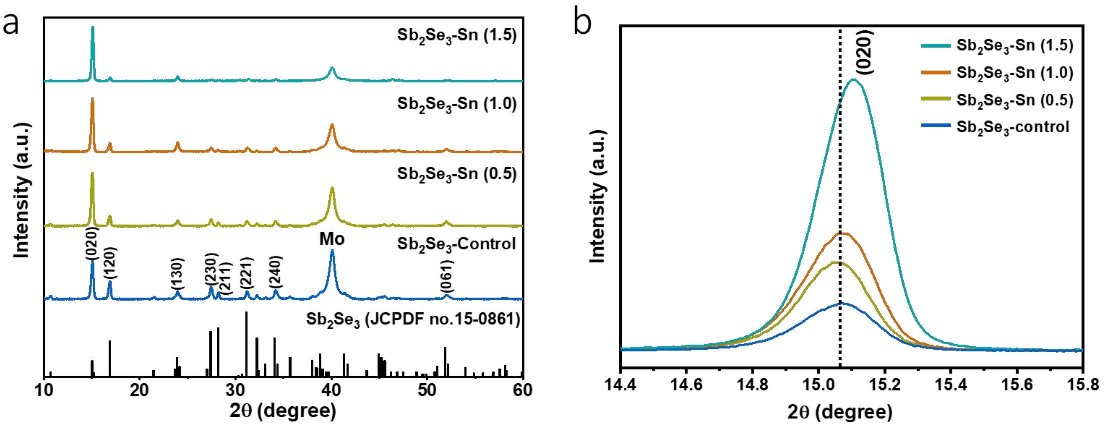

Figure S15. (a) XRD patterns of Sb_2Se_3-Control, Sb_2Se_3-Sn (0.5), Sb_2Se_3-Sn (1.0), and Sb_2Se_3-Sn (1.5) films. (b) Corresponding (020) peak with enlarged XRD patterns. Doping with the larger Sn^{2+} ion may cause the creation of selenium vacancies that partly compensate the charge, resulting in an overall shrinking of the lattice parameter.

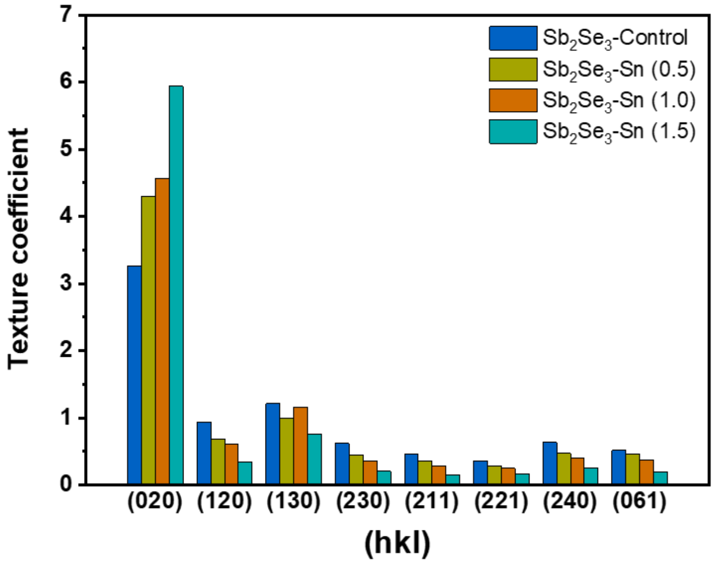

Figure S16. Texture coefficient of Sb_2Se_3-Control and Sb_2Se_3-Sn (0.5) films.

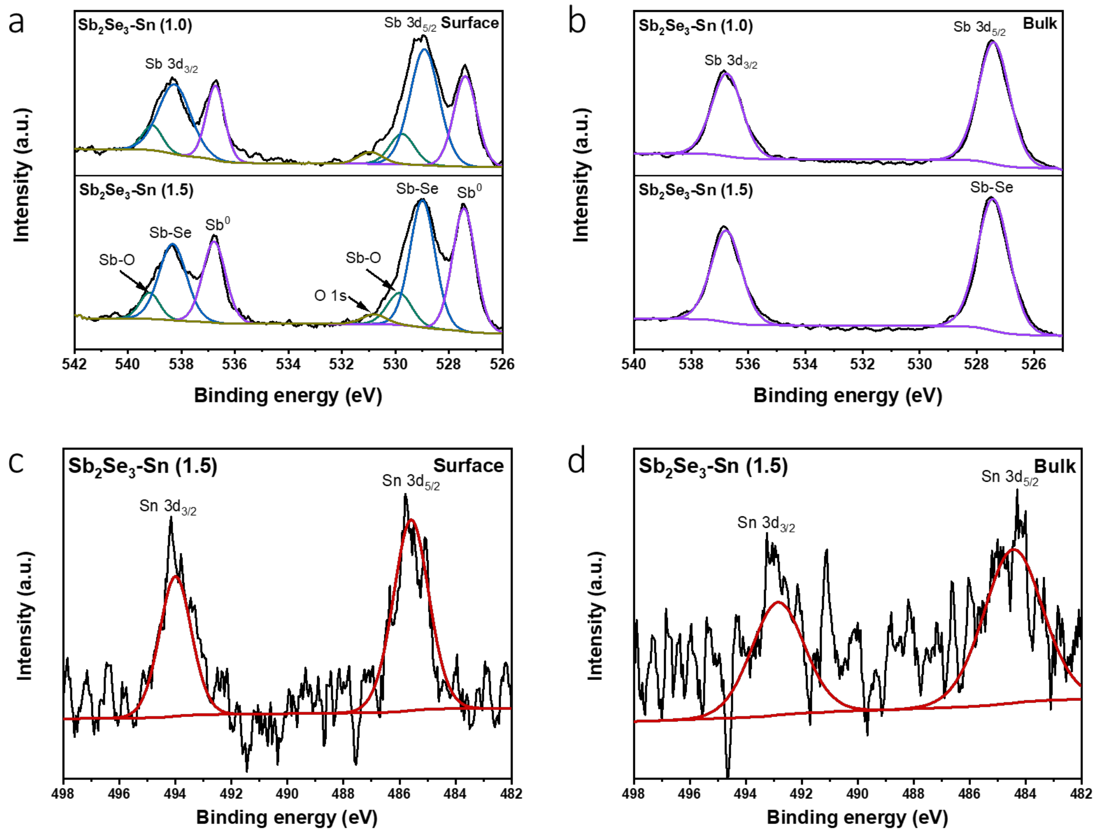

Figure S17. Sb 3d XPS spectra of Sb_2Se_3-Sn (1.0) and Sb_2Se_3-Sn (1.5) films (a) before and (b) after sputter etching. Sn 3d XPS spectra of Sb_2Se_3-Sn (1.5) film (c) before and (d) after sputter etching.

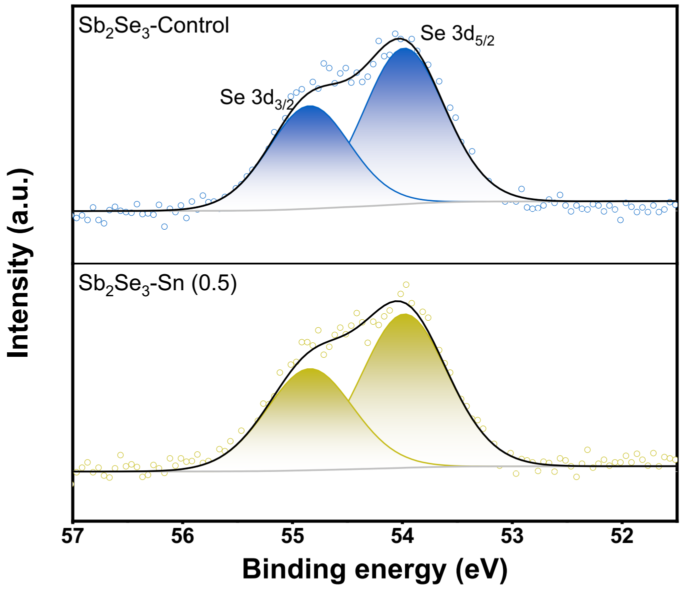

Figure S18. XPS spectra of Se 3d core level for Sb_2Se_3-Control and Sb_2Se_3-Sn (0.5) films.

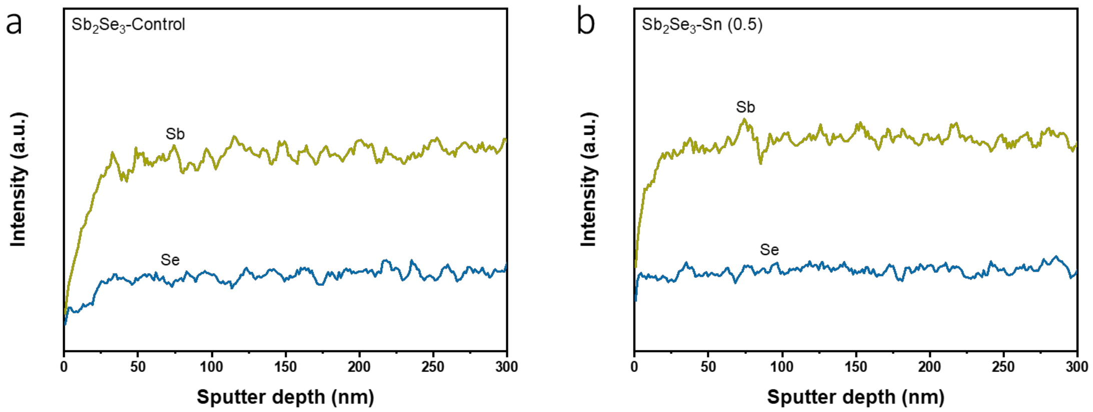

Figure S19. ToF-SIMS sputter depth profile for Sb and Se in the (a) Sb_2Se_3-Control and (b) Sb_2Se_3-Sn (0.5) films.

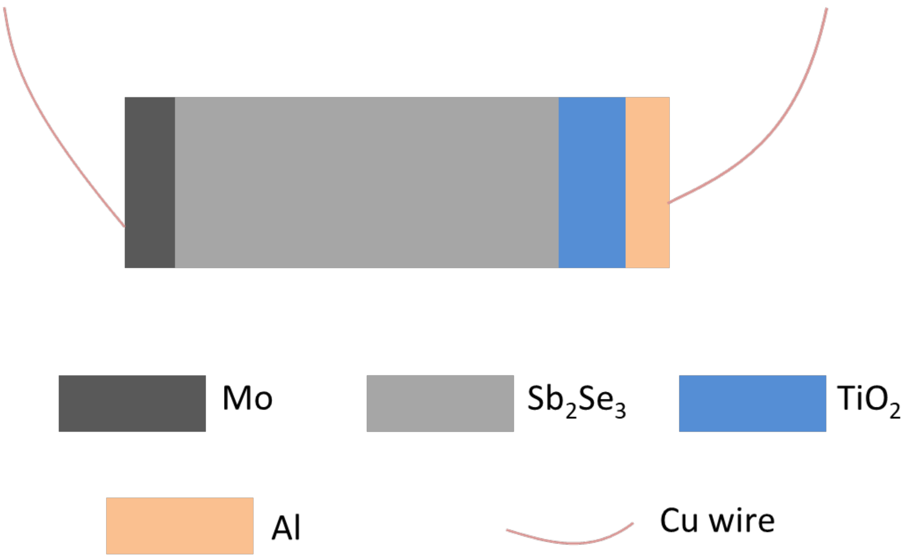

Figure S20. The device structure for CV and DLCP measurements.

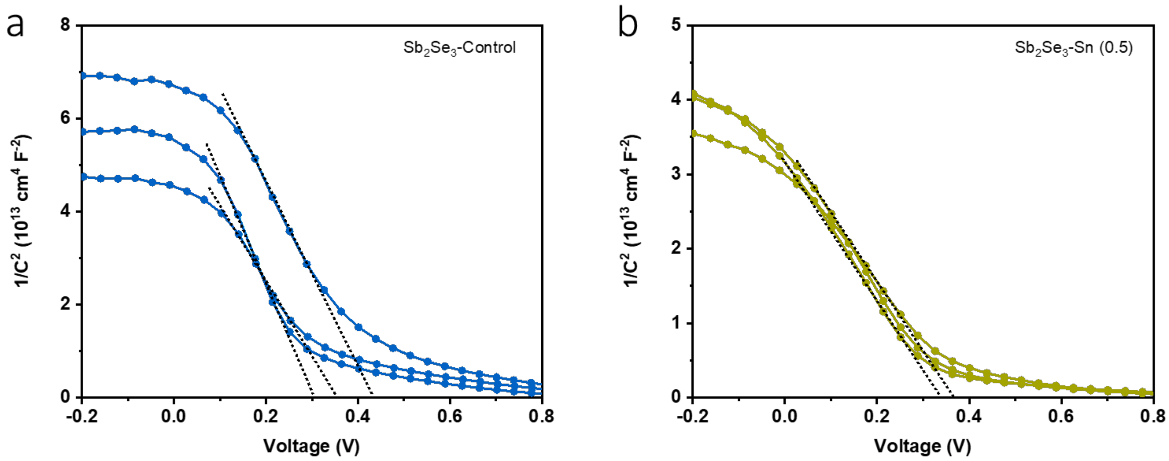

Figure S21. Mott-Schottky plots of (a) Sb_2Se_3-Control and (b) Sb_2Se_3-Sn (0.5) devices measured in 2-electrode mode. Three devices for each were measured.

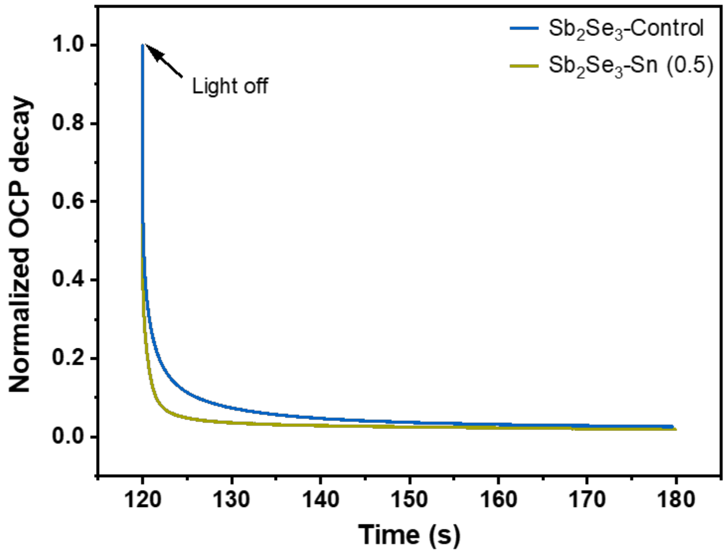

Figure S22. Normalized OCP decay of Sb_2Se_3-Control and Sb_2Se_3-Sn (0.5) photocathodes.

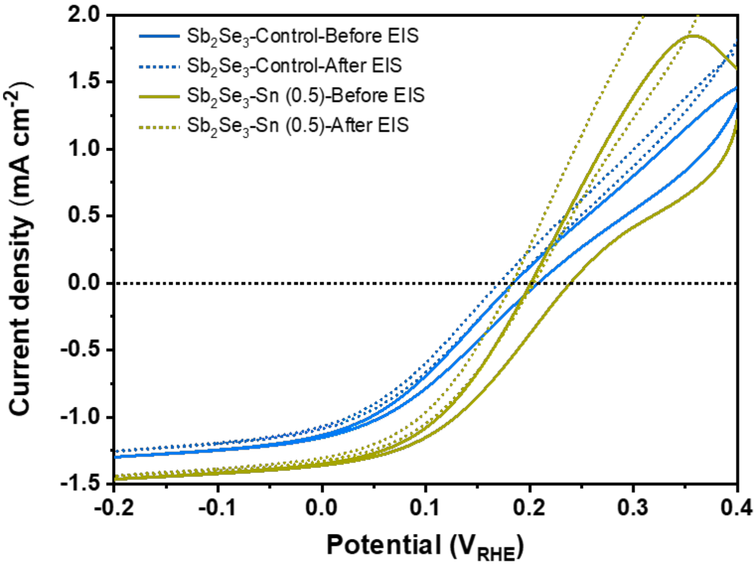

Figure S23. Cyclic voltammetry measurements of Sb_2Se_3-Control and Sb_2Se_3-Sn (0.5) photocathodes under 10% light illumination were recorded with a scan rate of 50 mV s^{-1} in 1 M H_2SO_4 solution (pH = 0) before and after EIS measurements.

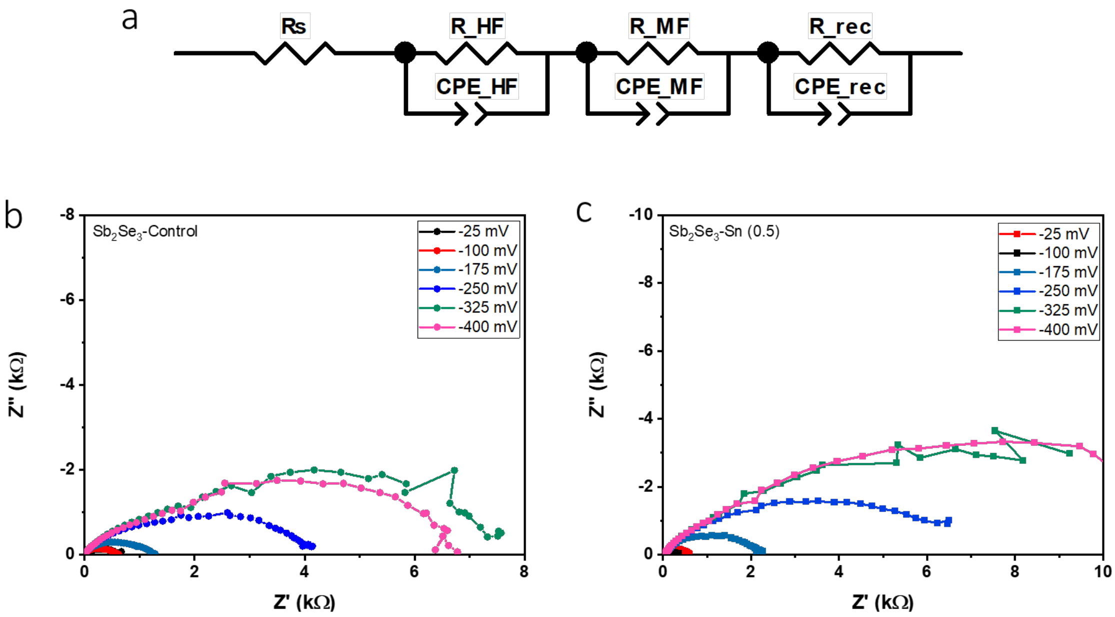

Figure S24. (a) The equivalent circuit used for the Nyquist plots fitting. Nyquist plots of the (b) Sb_2Se_3-Control and (c) Sb_2Se_3-Sn (0.5) photocathodes.

Table S1. Summary of the parameters obtained from CV, DLCP, and OCP measurements of Sb_2Se_3-Control and Sb_2Se_3-Sn (0.5) photocathodes.

Sample	Carrier density (N_{DLCP}) [cm^{-3}]	Interface defects density [cm^{-2}]	W_d [nm]	OCP [V]
Sb_2Se_3-Control	2.12×10^{16}	2.67×10^{12}	102	0.3
Sb_2Se_3-Sn (0.5)	3.77×10^{16}	8.64×10^{11}	76	0.32

Supplementary Note 1:

The formation of the right-hand branch of the U-shape was observed in the devices of Mo/Sb_2Se_3/TiO_2/Al and Mo/Sn-doped Sb_2Se_3/TiO_2/Al with increased reverse bias. This phenomenon is often interpreted as the result of non-uniform carrier density distribution within the absorber material. However, this conclusion would be hasty without considering the finite thickness (270 nm thickness) of the Sb_2Se_3 absorber in this case. The depletion region of the Sb_2Se_3/TiO_2 front junction extends toward the back contact with the increase of the applied reverse bias. At a certain applied potential, the depletion region from the Sb_2Se_3/TiO_2 front junction will reach the back contact, resulting in complete depletion of the Sb_2Se_3 absorber (the punch-through effect).[2] This effect can be found in the Mott-Schottky plots (Figure 4a), in

which the linear region (representing the space charge region) begins to vanish at 0.1 V for the Sb_2Se_3-control and 0.03 V for the Sb_2Se_3-Sn (0.5) devices. Because of the large number of free carriers at the back contact with Mo metal (~10^{23} cm^{-3}), there is no change of the depletion width on the p-side of the Sb_2Se_3/TiO_2 front junction with a further increase of reverse bias. While the depletion region may continue to expand on the n-side of the front junction (TiO_2/Al), the overall increase in depletion region width, such as a decrease of capacitance, is limited due to the higher carrier concentration in the atomic layer deposited TiO_2 (~2.6×10^{20} cm^{-3})[3] and Al (~10^{23} cm^{-3}). The manifestation of this punch-through effect in N_{CV} is a sharp increase from 10^{16-17} cm^{-3} to 10^{18-19} cm^{-3} when the depletion width approaches the thickness of the Sb_2Se_3 absorber. This explanation can be confirmed by the shape of the carrier density profile, in which the depletion width didn't significantly increase when the applied bias reached 0 V.

Supplementary Note 2:

Another way to evaluate the quality of Sb_2Se_3 is to study the photogenerated carrier lifetime as a function of OCP decay. Figure S20 displays the normalized transient OCP decay after stopping illumination. The slow decay of the Sb_2Se_3-Control on the order of 10s of seconds indicates the presence of trapped charge, likely at deep defects. that can serve as recombination centers for the photogenerated charges. A relatively fast decay is observed in the Sb_2Se_3-Sn (0.5) photocathode, indicating enhanced charge recombination when illumination is stopped.[1] Although it may seem counterintuitive at first sight why the better-performing device shows a faster OCP decay, several points can clarify this behavior. Firstly, the IPCE analysis reveals similar photon conversion at longer wavelengths within the bulk of both photocathodes, implying a similar trap density in the bulk of both Sb_2Se_3 photocathodes (see Figure 1d). However, in the short wavelength region, the Sb_2Se_3-Control photocathode exhibits lower IPCE conversion compared to the Sb_2Se_3-Sn (0.5) photocathode, indicating the primary difference between both devices lies in their interface between the absorber and TiO_2. This distinction is evident in the CV and DLCP measurements, which suggest a higher surface defect density observed in the Sb_2Se_3-Control device. Therefore, in the Sb_2Se_3-Control device, more charge carriers are trapped at the surface and are released at a slower rate, leading to decelerated OCP decay. The fast OCP decay observed in the Sb_2Se_3-Sn (0.5) device is probably also linked to the improved conductivity, facilitating rapid charge transport.

References

(1) Zhong, M.; Hisatomi, T.; Kuang, Y.; Zhao, J.; Liu, M.; Iwase, A.; Jia, Q.; Nishiyama, H.; Minegishi, T.; Nakabayashi, M.; Shibata, N.; Niishiro, R.; Katayama, C.; Shibano, H.; Katayama, M.; Kudo, A.; Yamada, T.; Domen, K. Surface Modification of CoO_x Loaded $BiVO_4$ Photoanodes with Ultrathin P-Type NiO Layers for Improved Solar Water Oxidation. *J. Am. Chem. Soc.* **2015**, *137* (15), 5053–5060.

(2) Li, J. V.; Halverson, A. F.; Sulima, O. V.; Bansal, S.; Burst, J. M.; Barnes, T. M.; Gessert, T. A.; Levi, D. H. Theoretical Analysis of Effects of Deep Level, Back Contact, and Absorber Thickness on Capacitance-Voltage Profiling of CdTe Thin-Film Solar Cells. *Sol. Energy Mater Sol. Cells* **2012**, *100*, 126–131.

(3) Moehl, T.; Suh, J.; Sévery, L.; Wick-Joliat, R.; Tilley, S. D. Investigation of (Leaky) ALD TiO_2 Protection Layers for Water-Splitting Photoelectrodes. *ACS Appl Mater Interfaces* **2017**, *9* (50), 43614–43622.
